# M6A-mediated upregulation of LINC00958 increases lipogenesis and acts as a nanotherapeutic target in hepatocellular carcinoma

**DOI:** 10.1186/s13045-019-0839-x

**Published:** 2020-01-08

**Authors:** Xueliang Zuo, Zhiqiang Chen, Wen Gao, Yao Zhang, Jinguo Wang, Junfeng Wang, Ming Cao, Juan Cai, Jindao Wu, Xuehao Wang

**Affiliations:** 1 Department of Gastrointestinal Surgery, The First Affiliated Hospital, Yijishan Hospital of Wannan Medical College, Wuhu, 241001 China; 2 Hepatobiliary Center, The First Affiliated Hospital of Nanjing Medical University, Key Laboratory of Liver Transplantation, Chinese Academy of Medical Sciences, NHC Key Laboratory of Liver Transplantation, Nanjing, 210029 China; 3 Key Laboratory of Non-coding RNA Transformation Research of Anhui Higher Education Institution (Wannan Medical College), Wuhu, 241001 China; 4 Department of Oncology, The First Affiliated Hospital of Nanjing Medical University, Nanjing, 210029 China; 5 Key Laboratory of Environment-Friendly Polymeric Materials of Anhui Province, School of Chemistry and Chemical Engineering, Anhui University, Hefei, 230601 China; 6 Department of Oncology, The First Affiliated Hospital, Yijishan Hospital of Wannan Medical College, Wuhu, 241001 China; 7 The Comprehensive Cancer Centre of Drum Tower Hospital, Medical School of Nanjing University & Clinical Cancer Institute of Nanjing University, Nanjing, 210008 China; 8 State Key Laboratory of Reproductive Medicine, Nanjing Medical University, Nanjing, 211166 China

**Keywords:** LINC00958, Hepatocellular carcinoma, N^6^-methyladenosine, Lipogenesis, HDGF

## Abstract

**Background:**

Long non-coding RNAs (lncRNAs) possess significant regulatory functions in multiple biological and pathological processes, especially in cancer. Dysregulated lncRNAs in hepatocellular carcinoma (HCC) and their therapeutic applications remain unclear.

**Methods:**

Differentially expressed lncRNA profile in HCC was constructed using TCGA data. LINC00958 expression level was examined in HCC cell lines and tissues. Univariate and multivariate analyses were performed to demonstrate the prognostic value of LINC00958. Loss-of-function and gain-of-function experiments were used to assess the effects of LINC00958 on cell proliferation, motility, and lipogenesis. Patient-derived xenograft model was established for in vivo experiments. RNA immunoprecipitation, dual luciferase reporter, biotin-labeled miRNA pull-down, fluorescence in situ hybridization, and RNA sequencing assays were performed to elucidate the underlying molecular mechanisms. We developed a PLGA-based nanoplatform encapsulating LINC00958 siRNA and evaluated its superiority for systemic administration.

**Results:**

We identified a lipogenesis-related lncRNA, LINC00958, whose expression was upregulated in HCC cell lines and tissues. High LINC00958 level independently predicted poor overall survival. Functional assays showed that LINC00958 aggravated HCC malignant phenotypes in vitro and in vivo. Mechanistically, LINC00958 sponged miR-3619-5p to upregulate hepatoma-derived growth factor (HDGF) expression, thereby facilitating HCC lipogenesis and progression. METTL3-mediated N^6^-methyladenosine modification led to LINC00958 upregulation through stabilizing its RNA transcript. A PLGA-based nanoplatform loaded with si-LINC00958 was developed for HCC systemic administration. This novel drug delivery system was controlled release, tumor targeting, safe, and presented satisfactory antitumor efficacy.

**Conclusions:**

Our results delineate the clinical significance of LINC00958 in HCC and the regulatory mechanisms involved in HCC lipogenesis and progression, providing a novel prognostic indicator and promising nanotherapeutic target.

**Electronic supplementary material:**

The online version of this article (10.1186/s13045-019-0839-x) contains supplementary material, which is available to authorized users.

## Introduction

With 841,080 new cases and 781,631 deaths annually, hepatocellular carcinoma (HCC) ranks the sixth most commonly diagnosed malignancy and the fourth leading cause of death worldwide [1]. Despite great efforts dedicated in the therapeutic strategies for HCC over the past years, including surgical resection, liver transplantation, and comprehensive therapy, the 5-year survival rate of HCC patients remains dismal. Therefore, elucidating the molecular mechanisms underlying HCC and determining novel molecular targets are essential to develop effective treatment modalities for this deadly malignancy.

Long non-coding RNAs (lncRNAs), a class of functional non-coding RNA transcripts > 200 nt in length, are engaged in diverse biological processes across every branch of life. Specific patterns of lncRNA expression coordinate cell differentiation, development, and pathogenesis. It has been widely recognized that many lncRNAs are dysregulated and play an important part in cancer progression [2]. In HCC, lncRNAs have been reported to affect various malignant phenotypes, such as cell proliferation, motility, and glucose metabolism reprogramming [3–5]. However, investigations of the involvement of lncRNAs in aberrant lipid metabolism in HCC are few. LncRNA-NEAT1 disrupts lipolytic enzyme ATGL-mediated lipolysis and drive HCC proliferation by binding miR-124-3p [6]. LncRNA HULC activates the acyl-CoA synthetase subunit ACSL1 in a miR-9-dependent manner to promote lipogenesis and function as an oncogene in hepatoma cells [7].

Long non-coding RNA 00958 (LINC00958) is originally identified as an oncogenic gene in bladder cancer by Seitz et al. [8]. Subsequent studies demonstrated that LINC00958 is upregulated in several other malignancies, including glioma [9], oral [10], gastric [11], pancreatic [12], and gynecological cancer [13, 14]. The involvement of LINC00958 in HCC has not yet been documented, prompting us to explore its biological functions and clinical value.

Polymeric nanoparticle (NP) platforms are emerged as promising carriers in cancer therapy by delivering a variety of drugs, including small interfering RNAs (siRNAs). NPs prevent siRNAs from rapid degradation, increase the drug concentration at tumor sites, and enable sustained release [15]. NPs formulated with poly(lactic acid/glycolic) (PLGA) copolymer are particularly attractive for clinical applications, due to their low immunogenicity, non-toxicity, biocompatibility, and biodegradability [16]. Poly(ethylene glycol) (PEG) is safe for clinical application and has been used in many Food and Drug Administration-approved medications including intravenous injections [17]. PEGylated PLGA NPs have been acknowledged as one of the best controlled release nanoplatforms for targeted drug delivery [18].

In the current study, we showed that LINC00958 was a lipogenesis-associated lncRNA that exacerbated HCC malignant phenotypes and independently predicted patient survival outcomes. Patient-derived xenograft (PDX) mouse models were adopted to evaluate the tumor-promoting role of LINC00958 in vivo. Mechanistically, METTL3-mediated N^6^-methyladenosine (m^6^A) induced the upregulation of LINC00958, which subsequently promoted HCC progression through the miR-3619-5p/HDGF axis. We developed a novel PLGA-based si-LINC00958 nanoplatform and evaluated its superiority for the treatment of HCC.

## Materials and methods

### Patients and tissue samples

Fresh tumor tissues and paired adjacent non-tumor samples were collected from 80 HCC patients who underwent surgical resection from January 2012 to December 2014 in the First Affiliated Hospital of Nanjing Medical University. The tissue samples were preserved in liquid nitrogen. All patients did not receive preoperative chemotherapy or radiotherapy and signed the written informed consents. This study was approved by the ethical review board of the First Affiliated Hospital of Nanjing Medical University.

### Fluorescence in situ hybridization (FISH)

Specific FISH probes to LINC00958 and miR-3619-5p were designed and synthesized by Servicebio (Wuhan, China). The hybridization was performed in HCC cells and tissues as previously reported [19]. All images were analyzed on a confocal laser scanning microscope (Leica Microsystems, Mannheim, Germany). The FISH probe sequences are shown as follows: LINC00958: 5′-TCCTCCCATGTTTTTGTCTTCCCTACCACC-3′; miR-3619-5p: 5′-GCTGCACCAGCCTGCCTGCTGA-3′.

### Lentivirus transfection and stable cell line construction

We purchased lentivirus overexpressing LINC00958 or HDGF, and small hairpin RNA (shRNA) targeting LINC00958 or METTL3 from Genechem (Shanghai, China). Lentiviruses were transfected into HCC cells with 5 mg/ml polybrene for 48 h. Stable cell clones were selected for 1 week using puromycin (5 μg/ml). The overexpression or knockdown efficiency was detected by RT-qPCR. The sequences used are provided as follows: sh1-LINC00958: 5′-GTACCCAAGTTATTCAGGATT-3′, sh2-LINC00958: 5′-GTGACTAGCTTAAACTAAATT-3′, sh3-LINC00958: 5′-GAGGTACCCAATAGTTTCATT-3′; sh-METTL3: 5′-GCCAAGGAACAATCCATTGTT-3′.

### RNA immunoprecipitation (RIP)

RIP assay was performed using a Magna RIP RNA-binding Protein Immunoprecipitation Kit (Millipore, Bedford, MA, USA) in accordance with the manufacturer’s protocol. Cells were isolated and lysed by RIP lysis buffer and incubated with antibodies against AGO2 (Abcam, Cambridge, MA, USA), or m^6^A (Synaptic Systems, Goettingen, German) at 4 °C overnight. IgG was used as negative control. The immunoprecipitated RNAs were eluted and analyzed by RT-qPCR.

### Biotin-labeled miRNA pull-down assay

Cells lysates were harvested 48 h after transfecting with 50 nM of biotin-labeled miRNAs (GeneCreate, Wuhan, China). Streptavidin-coupled Dynabeads (Invitrogen) were washed and resuspended in the buffer. Then an equal volume of the biotin-labeled miRNAs was added in the buffer. After incubating at room temperature for 10 min, the coated beads were separated with a magnet for 2 min and washed three times. The isolated RNAs were then subjected to RT-qPCR analysis.

### RNA sequencing

Total RNA was isolated from sh-NC (*n* = 3) and sh-LINC00958 (*n* = 3) HCCLM3 cells. RNA samples were analyzed by RNA sequencing (BGI, Shenzhen, China) based on the manufacturer’s protocols. Briefly, BGISEQ-500 platform was used to sequence the samples for subsequent generation of raw data. Genes significantly differentially expressed between sh-NC and sh-LINC00958 cells were selected based on fold change ≥ 2.0 and *P* ≤ 0.001 using the DEGseq method. Functional pathway analysis was conducted using KEGG pathway enrichment analysis.

### Oil Red O staining

HCC cells were fixed in 4% paraformaldehyde for 20 min and then permeabilized in 60% isopropanol for 10 s. Subsequently, cells were stained with Oil Red O working solution for 30 min at room temperature, washed three times with PBS, and photographed under a microscope. Oil Red O staining in frozen sections of HCC tissues were similarly performed.

### PDX mouse model

NOD/SCID and BALB/c mice were used for the establishment of the HCC PDX model. Briefly, we collected the primary HCC tissues from two patients after surgical resection and kept the specimens in iced culture medium supplemented with 1% penicillin/streptomycin. Then, the tissues were diced into 2–3-mm^3^ pieces and subcutaneously implanted into the flanks of NOD/SCID mice. When the xenografted tumors grew up to 1–2 cm^3^, we harvested the tissues from the mice bearing PDX tumors and cut the tissues into pieces. The tumor fragments were further implanted into BALB/c nude mice for the serial transplantation. When the tumor volume reached 50 mm^3^, we intratumorally injected recombinant lentivirus vectors into tumor tissues continuously for 20 days. Tumor weight and volume were recorded.

### Preparation of PLGA-PEG(si-LINC00958) NPs

We used the double emulsion solvent diffusion method for NP preparation as previously described [20]. si-LINC00958 was reconstituted in DEPC water and then mixed with spermidine (Sigma-Aldrich) at the N/P ratio (the ratio of polyamine amine groups to siRNA phosphate groups) of 8:1. The resultant mixture was incubated for 15 min at room temperature to form si-LINC00958/spermidine complex. PLGA-PEG-COOH (10 mg; DaiGang Biomaterial Co. Ltd., Jinan, China) was dissolved in 500 μl of dichloromethane (Aladdin Industrial Corp., Shanghai, China). Then, the above dichloromethane solution was added dropwise to si-LINC00958/spermidine complex with a probe sonicator (VCX 130; Sonics & Materials, Inc., Newtown, CT, USA) in an ice bath. The resultant primary emulsion was further added dropwise to 4 ml of an aqueous phase containing 2.5% polyvinyl alcohol (Aladdin Industrial Corp.) and emulsified using probe sonication for 1 min. The second emulsion was then stirred at room temperature for 4 h to evaporate the organic solvent. Subsequently, the NPs were collected by centrifugation for 15 min and washed twice with DEPC water.

We used dynamic light scattering (DLS) with a Nano Particle Analyzer (Zetasizer Nano ZSE, Malvern Instruments Ltd., UK) to investigate the size, zeta potential, and polydispersity index (PDI) of the NPs. A drop of the sample was placed onto a copper mesh and dried in room temperature to obtain transmission electron microscopy (TEM) images of the NPs. The siRNA encapsulated in PLGA was measured using UV spectrophotometry to determine the encapsulation efficiency as previously described [21].

### In vivo antitumor efficacy and toxicity evaluation of NPs

To investigate the suppressive effect of PLGA-PEG(si-LINC00958) NPs on HCC cell growth in vivo, PDX tumor models were created as described above. When the tumors developed to 50 mm^3^, PLGA-PEG(si-LINC00958) NPs or PLGA-PEG(siRNA control) NPs at a dose of 200 mg/kg were injected into the mice (*n* = 14 in each group) through the tail vein twice weekly. Treatment continued until 4 weeks later, at which point four mice in each group were sacrificed. Tumor weight and volume were recorded immediately. Tumors were subjected to subsequent RT-qPCR and western blotting analyses. The major organs, including the liver, kidney, lung, spleen, and heart, were harvested and fixed with 4% paraformaldehyde for further hematoxylin-eosin (H&E) examination. Blood alanine transaminase (ALT), aspartate transaminase (AST), creatinine (Cr), and blood urea nitrogen (BUN) were also analyzed. The remaining ten mice in each group were monitored for survival analysis with 10 weeks as the cutoff.

### Statistical analysis

SPSS 24.0 (IBM Corporation, Armonk, NY, USA) and GraphPad Prism 8.0 (GraphPad Software, La Jolla, CA, USA) were used to perform the statistical analysis. Data are shown as mean ± SEM of the mean. Two-sided Student’s *t* test was used to analyze the differences between groups. The differences of LINC00958 and HDGF expression levels between tumor and non-tumor specimens were evaluated by paired *t* test. Chi-square test was adopted to analyze the association of LINC00958 and METTL3 expression with clinicopathological features. Kaplan-Meier curve with log-rank test was used to compare the survival outcome, and Cox proportional hazards model was employed for multivariate survival analysis. Pearson’s correlation was performed to analyze the correlation between LINC00958, miR-3619-5p, METTL3, and HDGF levels. *P* value less than 0.05 was considered statistically significant.

Supplementary methods are described in Additional file 1.

## Results

### LINC00958 is highly expressed in HCC and predicts overall survival

**Fig. 1:**
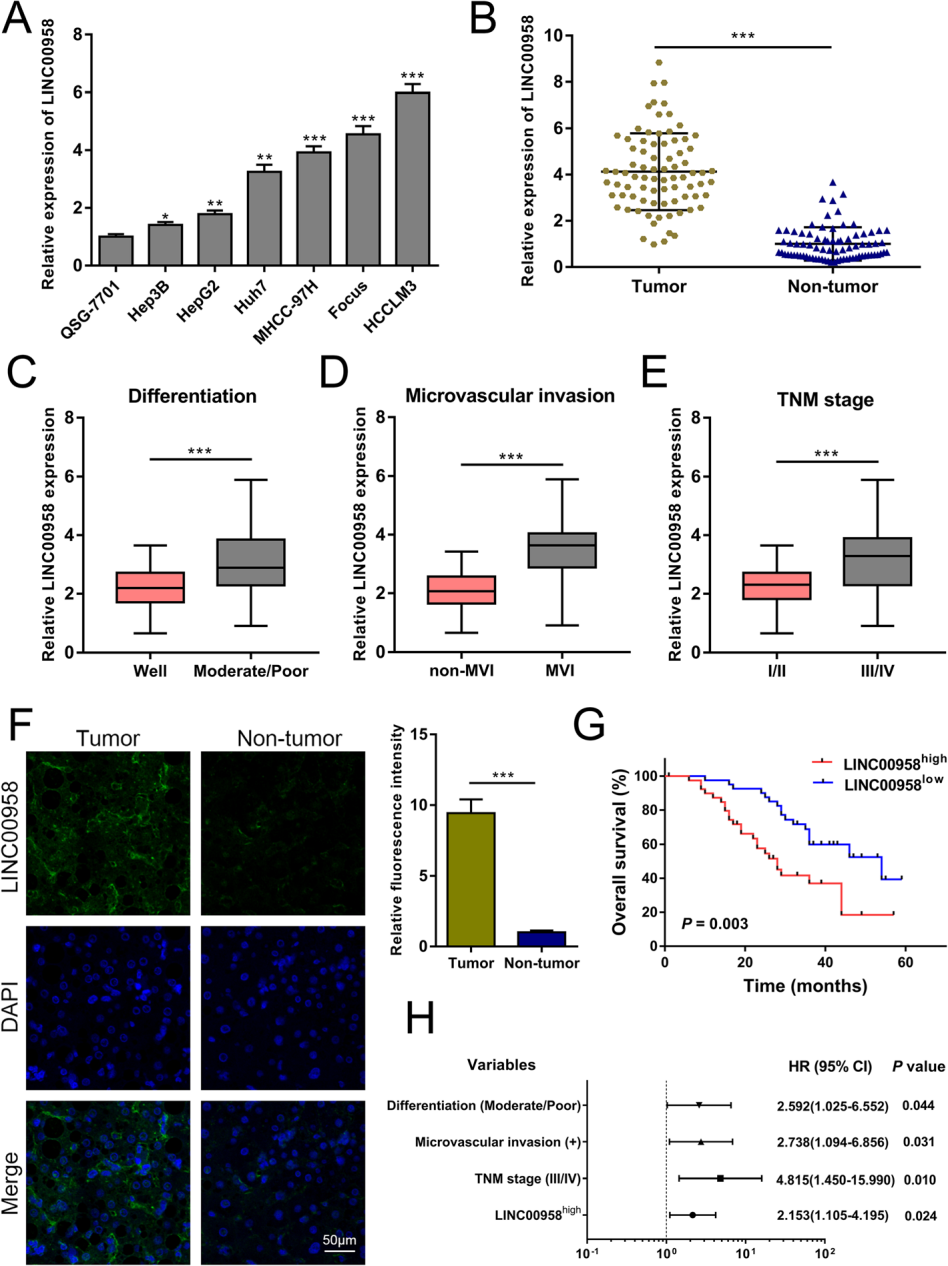
Highly expressed LINC00958 is associated with clinicopathological characteristics and independently predicts overall survival. **a** Expression levels of LINC00958 in normal human liver cell line QSG-7701 and six HCC cell lines (Hep3B, Hep2, Huh7, MHCC-97H, Focus, and HCCLM3) were examined using RT-qPCR. **b** The expression levels of LINC00958 in 80 paired HCC tissues and non-tumor specimens were determined using RT-qPCR. **c** LINC00958 expression levels were detected in patients with well tumor differentiation and those with moderate/poor differentiation (*n* = 80). **d** Expression levels of LINC00958 were compared between patients without microvascular invasion (MVI) and those with MVI (*n* = 80). **e** LINC00958 expression levels were assessed in the TNM stage I/II group and the TNM III/IV group (*n* = 80). **f** FISH was used to determine the expression of LINC00958 in HCC tissue and the paired non-tumor sample. Statistical data are shown. **g** Kaplan-Meier survival curves showing the effect of LINC00958 on overall survival (*P* = 0.003) (*n* = 80). **h** Multivariate analyses of the independent predictive factors for overall survival. Hazard ratio (HR) and the corresponding 95% confidence interval (CI) are shown. *P < 0.05, **P < 0.01, ***P < 0.001

With a stringent filter of logFC > 2.0 and *P* value < 0.005, we established the profile of differentially expressed lncRNAs in HCC based on TCGA data. As demonstrated in Additional file 2: Figure S1A, the expression levels of 441 lncRNAs were significantly altered in HCC tumor samples. LINC00958 was suggested to be upregulated in HCC (logFC = 4.092782 and *P* value = 4.44 × 10^− 7^). We then used the data retrieved from starBase platform and found a markedly higher expression of LINC00958 in HCC (Additional file 2: Figure S1B). To verify the bioinformatics results, we performed RT-qPCR to quantify the expression levels of LINC00958 in normal human liver cell line QSG-7701 and six HCC cell lines. As shown in Fig. 1a, LINC00958 was highly expressed in HCC cell lines. Furthermore, we examined LINC00958 expression in 80 paired HCC tissues and non-tumor specimens. LINC00958 was remarkably overexpressed in HCC tissues (Fig. 1b), especially in those with moderate/poor differentiation, microvascular invasion, and TNM III/IV stage (Fig. 1c–e). FISH assay verified the overexpression of LINC00958 in HCC tissues compared to the non-tumor samples (Fig. 1f).

To investigate the clinical significance of LINC00958 in HCC, we classified the enrolled 80 patients into LINC00958^high^ and LINC00958^low^ groups based on the median expression value (2^−△△Ct^ = 0.134). As indicated in Additional file 3: Table S1, high LINC00958 expression was associated with tumor differentiation (*P* = 0.019), tumor size (*P* = 0.025), microvascular invasion (*P* = 0.014), and TNM stage (*P* = 0.013). Bioinformatics prediction implied a correlation between LINC00958 expression status and patient survival (*P* = 0.014; Additional file 2: Figure S1C), and we confirmed that patients with high LINC00958 expression had poorer overall survival than those with low LINC00958 expression (*P* = 0.003; Fig. 1g). Multivariate Cox regression analysis showed that LINC00958 was an independent prognostic factor for HCC patients [hazard ratio (HR) 2.153, 95% confidence interval (CI) 1.105–4.195, *P* = 0.024; Fig. 1h and Additional file 4: Table S2].

### LINC00958 is required for malignant behaviors in HCC cells

**Fig. 2:**
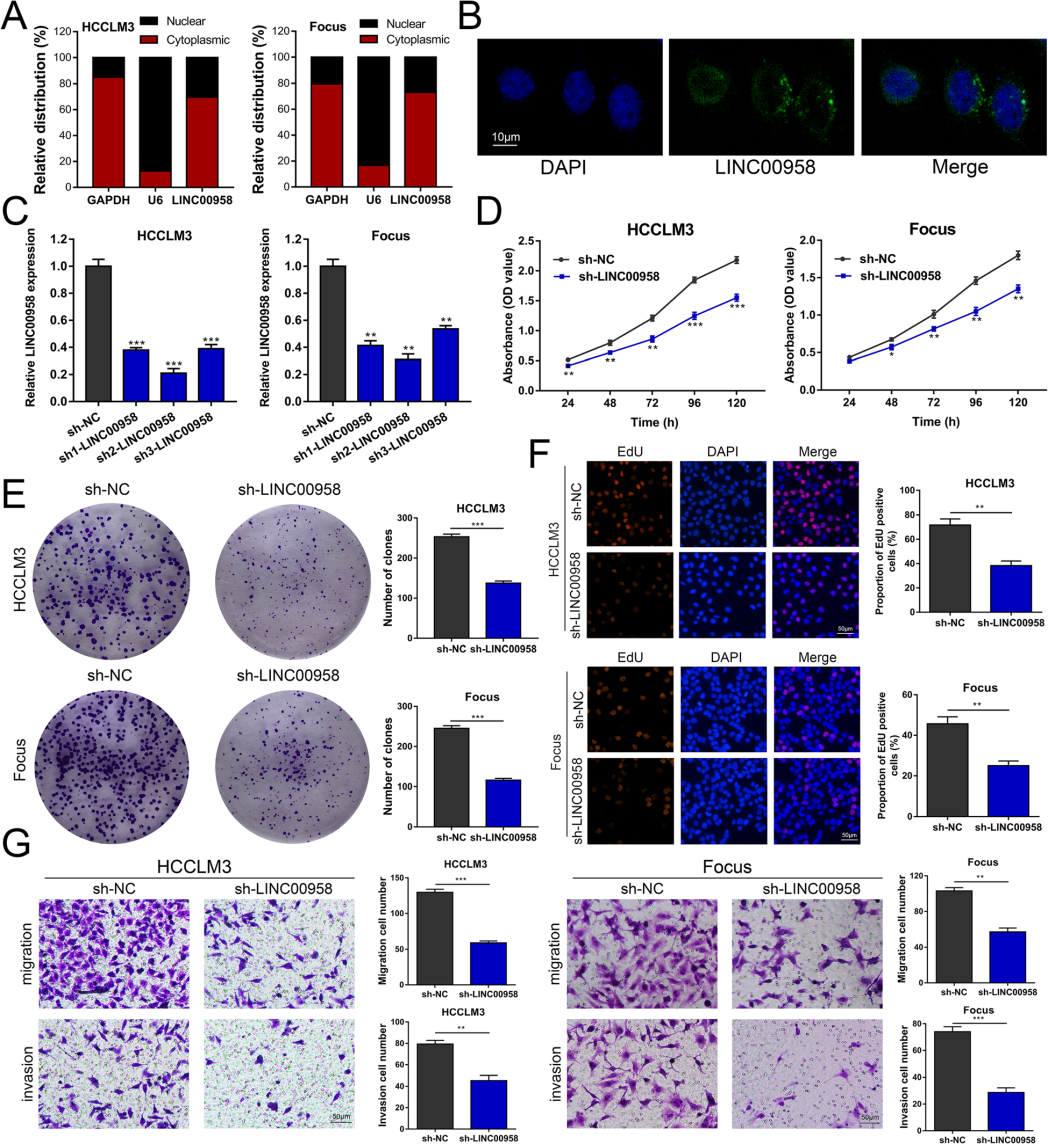
Knockdown of LINC00958 inhibits HCC proliferation, migration, and invasion in vitro. **a** Levels of LINC00958 from the nuclear and cytoplasmic fractions of HCCLM3 and Focus cells were evaluated using RT-qPCR. GAPDH and U6 were used as positive control for the cytoplasmic and nuclear fraction, respectively. **b** FISH was performed to determine the subcellular distribution of LINC00958 in HCCLM3 cells. **c** The expression of LINC00958 was knocked down using three shRNAs in HCCLM3 and Focus cells. **d** CCK-8 assays were performed to assess the cell proliferation in LINC00958-silenced HCC cells. **e** Colony formation assays showed the clone numbers in HCC cells with LINC00958 knockdown. **f** EdU assays were performed to assess the proliferative ability of HCCLM3 and Focus cells with LINC00958 knockdown. **g** Transwell assays were conducted to examine the effects of LINC00958 knockdown on HCC cell migration and invasion. *P < 0.05, **P < 0.01, ***P < 0.001

We performed RT-qPCR and FISH assays and showed that LINC00958 was predominantly located in cytoplasm (Fig. 2a, b). To evaluate the function of LINC00958 in HCC, we stably knocked down the expression of LINC00958 by three shRNAs (sh1-LINC00958, sh2-LINC00958, and sh3-LINC00958) in HCCLM3 and Focus cells. As shown in Fig. 2c, sh2-LINC00958 exhibited the most evident knockdown effect and was chosen for the subsequent experiments. CCK-8 assays demonstrated that silencing LINC00958 significantly reduced the proliferative capabilities of HCCLM3 and Focus cells (Fig. 2d). The inhibitory effects of LINC00958 knockdown on HCC cell proliferation were further confirmed by colony formation and EdU assays (Fig. 2e, f). Transwell assays showed that HCC cells transfected with sh-LINC00958 presented a markedly decreased migration and invasion abilities (Fig. 2g). In addition, we overexpressed LINC00958 via lentivirus in Hep3B and HepG2 cells (Additional file 5: Figure S2A). Based on the results from CCK-8 assays, we observed increased cell growth rates in HCC cells with LINC00958 overexpression (Additional file 5: Figure S2B). We also performed colony formation and EdU assays and showed that LINC00958 overexpression significantly elevated the proliferation of Hep3B and HepG2 cells (Additional file 5: Figure S2C-D). LINC00958 overexpression greatly promoted cell migration and invasion abilities in HCC (Additional file 5: Figure S2E). Collectively, these data indicated that LINC00958 facilitates HCC proliferation and migration in vitro.

### LINC00958 targets miR-3619-5p to exert its tumor-promoting effects in HCC

**Fig. 3:**
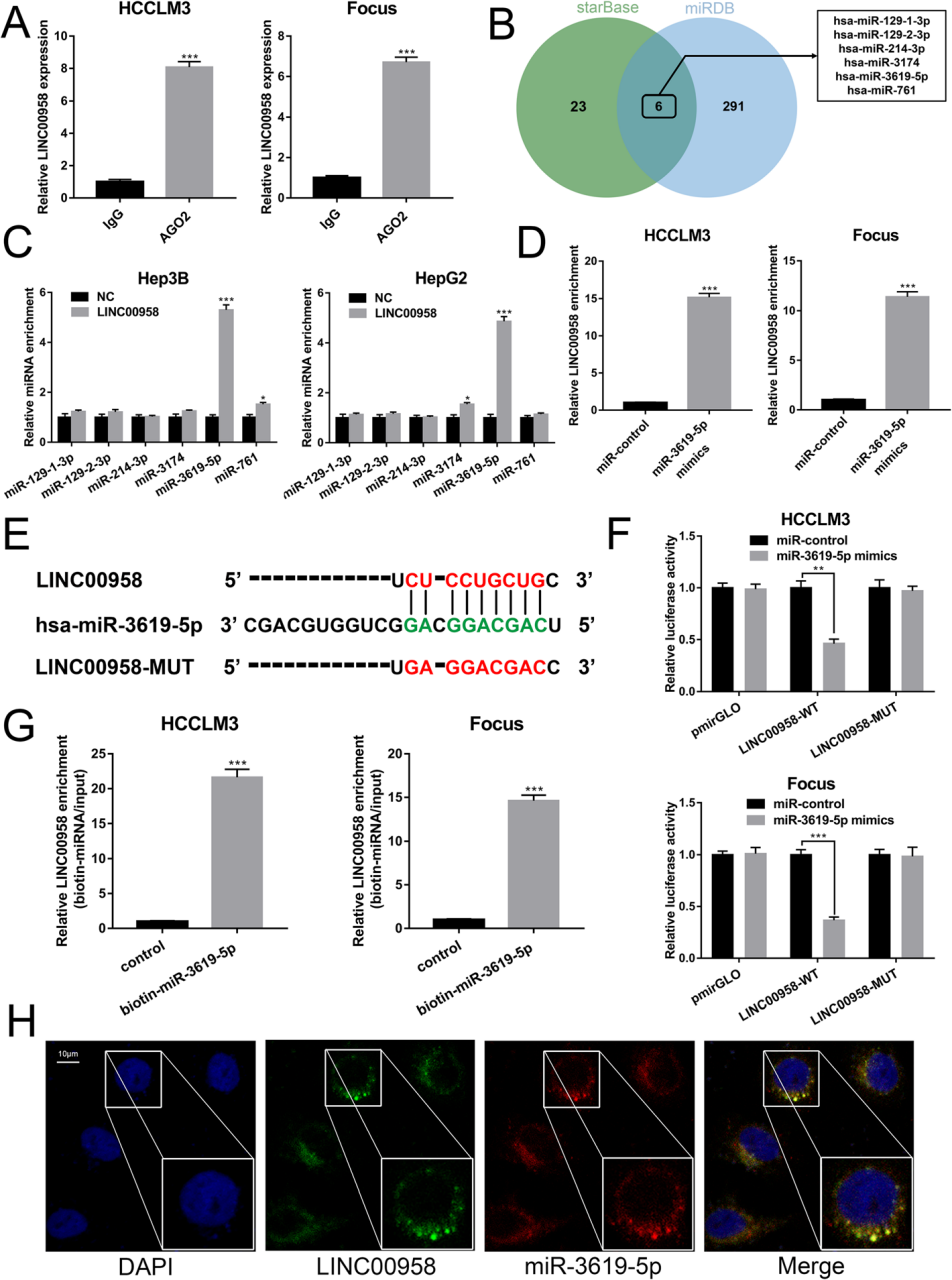
LINC00958 targets miR-3619-5p to exert its tumor-promoting effects in HCC. **a** RIP assay for AGO2 was conducted to detect the levels of endogenous LINC00958 in the AGO2 IP pellet. **b** A total of six miRNAs were predicted to harbor complementary sequences to LINC00958 according to starBase and miRDB databases. **c** AGO2-RIP assays showed the enrichment of the predicted six miRNAs in Hep3B and HepG2 cells with LINC00958 overexpression or NC. **d** Enrichment of LINC00958 in HCCLM3 and Focus cells transfected with miR-3619-5p mimics or miR-control. **e** Putative binding sequence between LINC00958 and miR-3619-5p. **f** Luciferase reporters containing WT or MUT LINC00958 transcript as well as blank pmirGLO were co-transfected with miR-3619-5p mimics or miR-control in HCCLM3 and Focus cells. Luciferase activity was determined using dual luciferase reporter system. **g** Enrichment of LINC00958 pulled down by biotin-miR-3619-5p or negative control. **h** FISH results showing the colocalization of LINC00958 and miR-3619-5p in cytoplasm in HCC cells. **P < 0.01, ***P < 0.001

Given the cytoplasmic distribution of LINC00958, we hypothesized that LINC00958 might exert its effects via targeting miRNAs. As demonstrated in Fig. 3a, the results from RIP assays using an anti-AGO2 antibody showed that endogenous LINC00958 was preferentially enriched in the AGO2 IP pellet compared to control IgG IP pellet. To explore the underlying regulatory mechanism of LINC00958, we used starBase and miRDB databases and found six miRNAs with potential complementary binding sequences (Fig. 3b and Additional file 6: Supplementary Material 1). AGO2-RIP assays showed that miR-3619-5p was the highest enriched miRNA in the LINC00958-overexpressed group compared to the negative control (NC) group (Fig. 3c) and LINC00958 enrichment was much higher in the miR-3619-5p mimics group compared to the miR-NC group (Fig. 3d). These data suggested that LINC00958 and miR-3619-5p existed in the same RNA-induced silencing complex. Furthermore, we generated a mutant sequence of LINC00958 that could not bind miR-3619-5p for the subsequent luciferase reporter assays (Fig. 3e). As demonstrated in Fig. 3f, miR-3619-5p mimics significantly decreased the luciferase activity in HCC cells transfected with the wildtype LINC00958 sequence, whereas the luciferase activity was not obviously altered in HCC cells transfected with the mutant LINC00958. Subsequently, biotin-labeled miRNA pull-down assays showed significantly increased LINC00958 interaction in the HCC cells transfected with biotin-labeled miR-3619-5p compared to that in the control (Fig. 3g). FISH assays revealed the colocalization of LINC00958 and miR-3619-5p in the cytoplasm in HCC cells (Fig. 3h).

To confirm that LINC00958 exerted the tumor-promoting effects via miR-3619-5p, we upregulated the expression of miR-3619-5p in LINC00958-overexpressed Hep3B cells (Additional file 7: Figure S3A) and conducted rescue experiments. As indicated in Additional file 7: Figure S3B-C, the elevated proliferation ability in LINC00958-overexpressed Hep3B cells was counteracted by miR-3619-5p mimics. Similarly, miR-3619-5p overexpression could rescue the increased cell migration and invasion in Hep3B cells overexpressing LINC00958 (Additional file 7: Figure S3D).

### HDGF, a direct target of miR-3619-5p, is crucial for the function of LINC00958

**Fig. 4:**
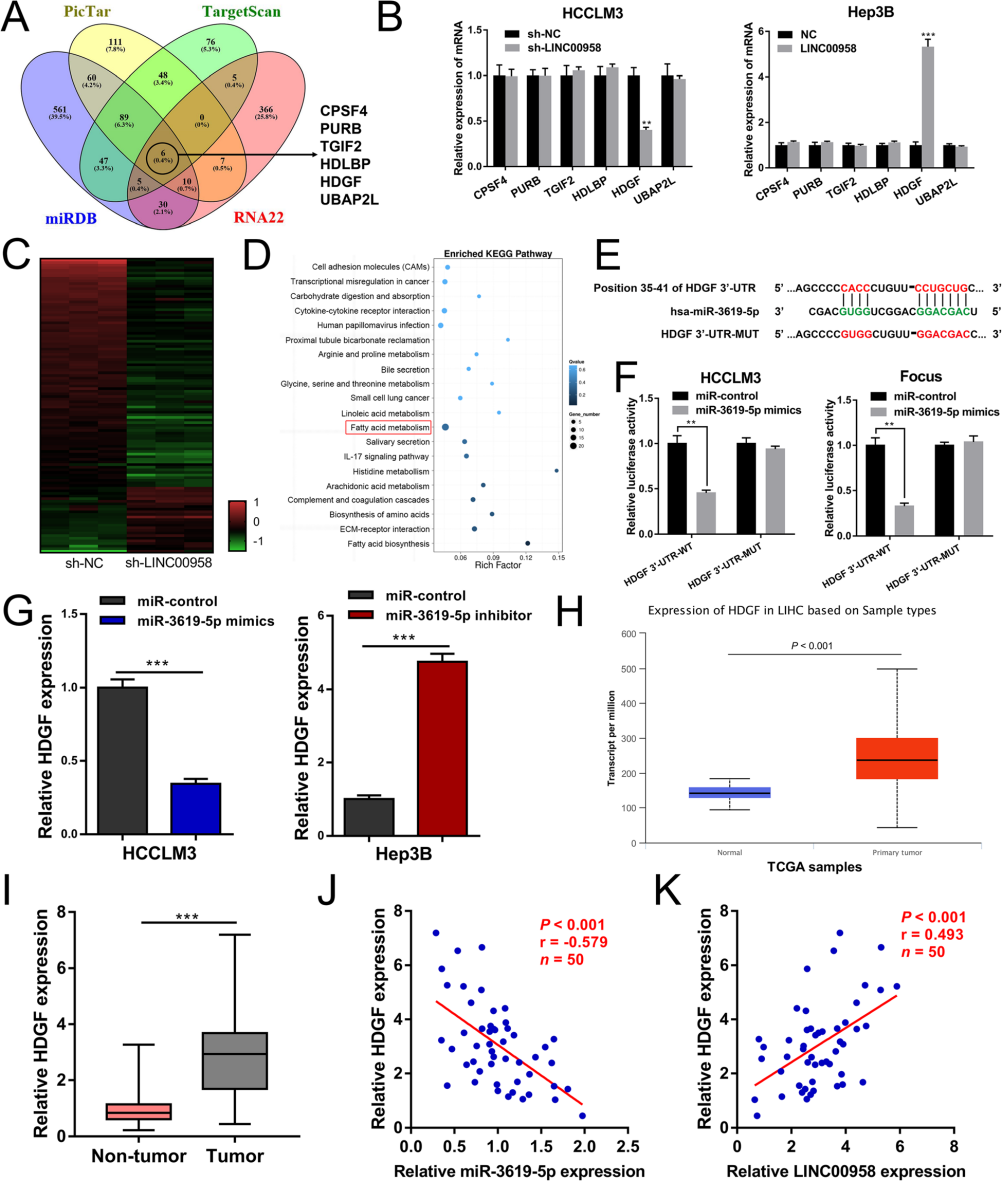
HDGF is a direct target gene of miR-3619-5p. **a** Venn diagram showing six putative miR-3619-5p target genes predicted by four different algorithms (PicTar, TargetScan, miRDB, and RNA22). **b** RT-qPCR analysis showed that HDGF was downregulated in LINC00958-silenced HCCLM3 cells and upregulated in LINC00958-overexpressed Hep3B cells. **c** Heat map showing the differentially expressed genes modulated by LINC00958 knockdown. **d** Enriched KEGG pathway analysis showing the most enriched pathways. **e** Putative binding sequence of miR-3619-5p in the 3′-UTR of HDGF. **f** Dual luciferase reporter assay revealed that miR-3619-5p could bind to the 3′-UTR of HDGF. **g** Expression levels of HDGF were detected by RT-qPCR in miR-3619-5p-overexpressed HCCLM3 cells or miR-3619-5p-silenced Hep3B cells. **h** TCGA data suggested that the expression level of HDGF was upregulated in liver cancer samples. **i** RT-qPCR results showing the expression levels of HDGF in 50 paired HCC tissues and non-tumor specimens. **j** Correlation analysis showing a negative correlation between miR-3619-5p and HDGF expression (*P* = 0.011). **k** Expression level of LINC00958 was positively correlated with HDGF expression level (*P* = 0.002)

To investigate the targets of miR-3619-5p regulated by LINC00958, we performed bioinformatics analysis using four different algorithms including PicTar, TargetScan, miRDB, and RNA22 (Additional file 8: Supplementary Material 2). Figure 4a shows the overlapping target genes of miR-3619-5p. Expression analysis yielded that only hepatoma-derived growth factor (HDGF) was downregulated in HCCLM3 cells upon LINC00958 knockdown and upregulated in Hep3B cells upon LINC00958 overexpression (Fig. 4b). Furthermore, RNA sequencing was performed to identify the differentially expressed genes in LINC00958-silenced HCC cells. Using DEGseq method, we found that 429 genes were downregulated and 134 genes were upregulated after LINC00958 knockdown (Fig. 4c). Enriched KEGG pathway analysis on RNA sequencing data was shown in Fig. 4d. Notably, HDGF was found downregulated by 2.63-fold after LINC00958 knockdown. We then mutated the miR-3619-5p binding site of the 3′-UTR of HDGF to construct the pmirGLO-HDGF 3′-UTR-MUT vector (Fig. 4e). Luciferase reporter assays indicated that miR-3619-5p mimics significantly decreased the luciferase activity of pmirGLO-HDGF 3′-UTR-WT, but had no effect on the activity of pmirGLO-HDGF 3′-UTR-MUT (Fig. 4f). We then investigated the expression levels of HDGF in HCC cells transfected with miR-3619-5p mimics or inhibitor. In HCCLM3 cells, miR-3619-5p mimics led to a decreased expression level of HDGF. Compared with the control cells, Hep3B cells transfected with miR-3619-5p inhibitor presented a higher level of HDGF expression (Fig. 4g). As demonstrated in both TCGA data (Fig. 4h) and our RT-qPCR results (Fig. 4i), HDGF was remarkably upregulated in HCC tissues. Correlation analysis suggested a negative correlation between the level of miR-3619-5p and HDGF expression level in HCC tissue specimens (Fig. 4j). In addition, the expression level of LINC00958 was positively correlated with HDGF expression level (Fig. 4k).

To confirm that HDGF was the downstream target of LINC00958-mediated HCC progression, we performed the subsequent functional rescue assays. We first overexpressed HDGF in HCCLM3 sh-LINC00958 cells and verified the overexpression efficiency by RT-qPCR and western blotting (Additional file 9: Figure S4A-B). Based on the CCK-8 and EdU assays, we found that HDGF overexpression could rescue the suppressed proliferative capability in HCCLM3 sh-LINC00958 cells (Additional file 9: Figure S4C-D). Transwell assays showed that the inhibited cell motility was restored after overexpressing HDGF in HCCLM3 sh-LINC00958 cells (Additional file 9: Figure S4E). Together, these data indicated the tumor-promoting role of the LINC00958/miR-3619-5p/HDGF axis in HCC.

### LINC00958 positively correlates with lipogenesis in HCC cells

**Fig. 5:**
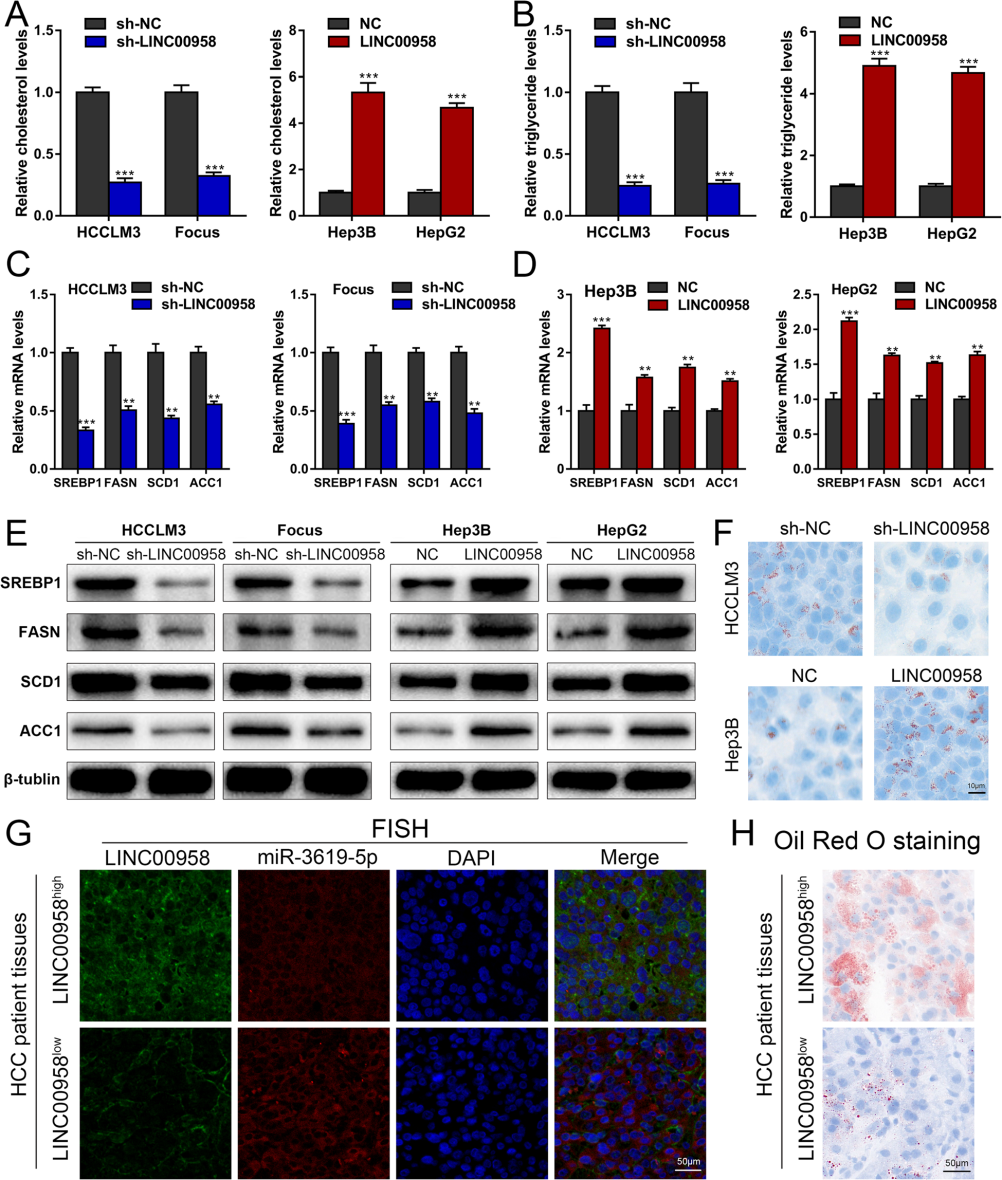
LINC00958 positively correlates with lipogenesis in HCC cells. **a** Cellular cholesterol levels were assessed in HCC cells with LINC00958 knockdown or overexpression. **b** Cellular levels of triglyceride were assessed in HCC cells with LINC00958 knockdown or overexpression. **c** Expression levels of SREBP1, FASN, SCD1, and ACC1 in HCCLM3 and Focus cells with LINC00958 knockdown were detected using RT-qPCR. **d** Expression levels of SREBP1, FASN, SCD1, and ACC1 in Hep3B and HepG2 cells with LINC00958 overexpression were examined using RT-qPCR. **e** Western blotting was used to determine the levels of SREBP1, FASN, SCD1, and ACC1 in LINC00958-silenced HCCLM3 and Focus cells and LINC00958-overexpressed Hep3B and HepG2 cells. **f** Oil Red O staining showing the lipid droplets in HCC cells with LINC00958 knockdown or overexpression. **g** Representative FISH images showing the expression levels of LINC00958 and miR-3619-5p in LINC00958^high^ and LINC00958^low^ HCC patient tissues. **h** Oil Red O staining was performed to show the lipid droplets in tissue samples of LINC00958^high^ and LINC00958^low^ HCC patients. **P < 0.01, ***P < 0.001

Mounting evidence indicates that abnormal lipid metabolism plays crucial parts in HCC development [22–24], and HDGF was recently reported to be a lipogenesis-associated gene in tumorigenesis [25]. We started to explore whether LINC00958 could affect lipogenesis in HCC cells. Pathway enrichment results also revealed that fatty acid metabolism was among the top canonical pathways (Fig. 4d). As shown in Fig. 5a, b, HCCLM3 and Focus cells with LINC00958 knockdown exhibited reduced cellular levels of cholesterol and triglyceride, whereas Hep3B and HepG2 cells overexpressing LINC00958 presented higher cholesterol and triglyceride levels compared with the control cells. HCCLM3 and Focus cells with LINC00958 knockdown showed decreased mRNA levels of several key enzymes in lipogenesis, including SREBP1, FASN, SCD1, and ACC1 (Fig. 5c). In contrast, Hep3B and HepG2 cells with LINC00958 overexpression showed increased SREBP1, FASN, SCD1, and ACC1 mRNA levels (Fig. 5d). Western blotting results confirmed that LIN00958 was positively correlated with the protein levels of SREBP1, FASN, SCD1, and ACC1 in HCC cells (Fig. 5e). To further examine such positive correlation of LINC00959 with lipogenesis in HCC cells, we performed Oil Red O staining in HCC cells with LINC00958 knockdown or overexpression. Lipid droplets comprising mainly triglycerides and sterol esters, as indicated by Oil Red O staining, were less abundant in HCCLM3 cells with LINC00958 knockdown than in the control cells. Conversely, more lipid droplets were observed in Hep3B cells with LINC00958 overexpression than in the control cells (Fig. 5f). Furthermore, more lipid droplets were detected by Oil Red O staining in HCC patient samples with high LINC00958 level compared to those with low LINC00958 expression level (Fig. 5g, h).

To verify that LINC00958 promoted lipogenesis through miR-3619-5p, we performed the subsequent rescue experiments. As demonstrated in Additional file 10: Figure S5A-B, the elevated cholesterol and triglyceride levels in LINC00958-overexpressed Hep3B cells were ameliorated by miR-3619-5p mimics. High SREBP1 mRNA and protein levels in LINC00958-overexpressed Hep3B cells were counteracted by transfecting miR-3619-5p mimics (Additional file 10: Figure S5C-D). Oil Red O staining results suggested that miR-3619-5p overexpression restored the elevated lipid droplets in LINC00958-overexpressed Hep3B cells (Additional file 10: Figure S5E). Likewise, we investigated the effects of HDGF overexpression on lipogenesis in LINC00958-silenced HCCLM3 cells. As shown in Additional file 11: Figure S6A-E, the inhibited cellular cholesterol and triglyceride levels, SREBP1 levels, and lipid droplet levels in LINC00958-silenced HCCLM3 cells were rescued by HDGF overexpression, suggesting that HDGF was the downstream effector of LINC00958-mediated lipogenesis. Together, these data supported that LINC00958 promoted lipogenesis in HCC through the miR-3619-5p/HDGF signaling pathway.

### LINC00958 facilitates HCC growth in vivo

**Fig. 6:**
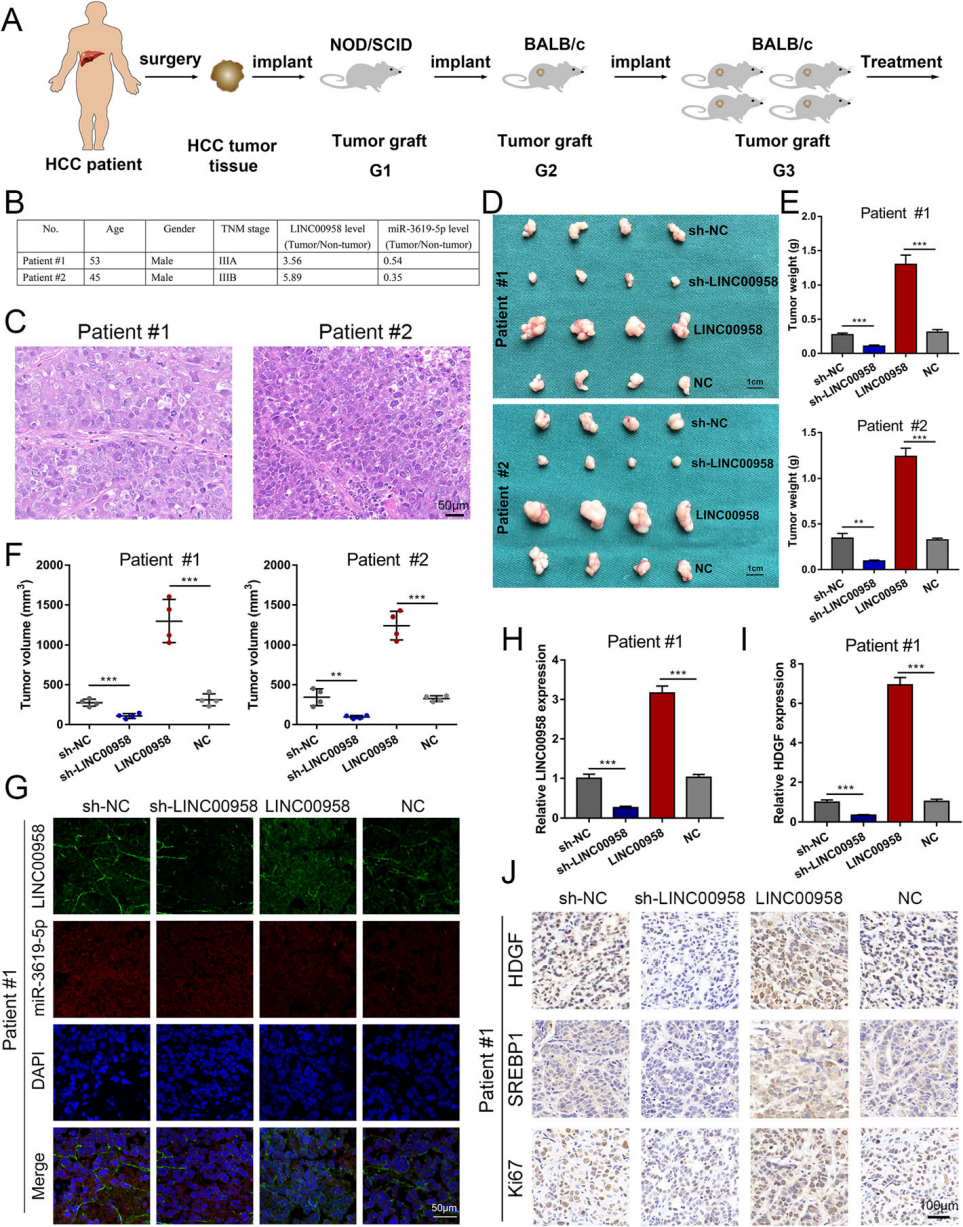
LINC00958 facilitates HCC growth in vivo. **a** A graphic illustration of the construction of HCC PDX mouse models. **b** The donor patients were clinically characterized. **c** The engrafted tumors were histopathologically analyzed. **d** The engrafted tumors in the sh-NC, sh-LINC00958, LINC00958, NC groups were harvested. **e** Tumor weight was recorded in the engrafted tumors. **f** Tumor volume was measured in the engrafted tumors. **g** FISH results showing the expression levels of LINC00958 in the engrafted tumors. **h** The expression level of LINC00958 was examined in the engrafted tumors by RT-qPCR. **i** The expression level of HDGF was assessed in the engrafted tumors by RT-qPCR. **j** The expression levels of HDGF, SERBP1, and Ki67 were determined in PDX tumor tissues using immunohistochemistry. ***P < 0.001

PDX mouse models were utilized to investigate the effects of LINC00958 on HCC growth in vivo. We intratumorally injected recombinant sh-NC, sh-LINC00958, LINC00958, and NC on four groups of PDX mice, respectively (Fig. 6a). Clinical characterization of the donor patients is presented in Fig. 6b. We histopathologically analyzed the engrafted tumors using H&E staining (Fig. 6c). As indicated in Fig. 6d–f, we found that LINC00958 knockdown resulted in a blunted tumor growth in terms of tumor weight and volume, whereas LINC00958 overexpression accelerated tumor growth. We then determined the expression levels of LINC00958 in the four groups of PDX tumors by FISH and RT-qPCR. As shown in Fig. 6g, h, decreased LINC00958 levels were detected in the sh-LINC00958 group, whereas elevated LINC00958 levels were observed in the LINC00958 group. The expression level of HDGF was found downregulated in the sh-LINC00958 group and upregulated in the LINC00958 group (Fig. 6i). The expression levels of HDGF, SERBP1, and Ki67 in the PDX tumors were then examined by immunohistochemistry. As presented, downregulated HDGF, SERBP1, and Ki67 expression levels were detected in the sh-LINC00958 group, while upregulated levels of HDGF, SERBP1, and Ki67 were found in the LINC00958 group (Fig. 6j).

### m^6^A modification is associated with LINC00958 upregulation in HCC cells

**Fig. 7:**
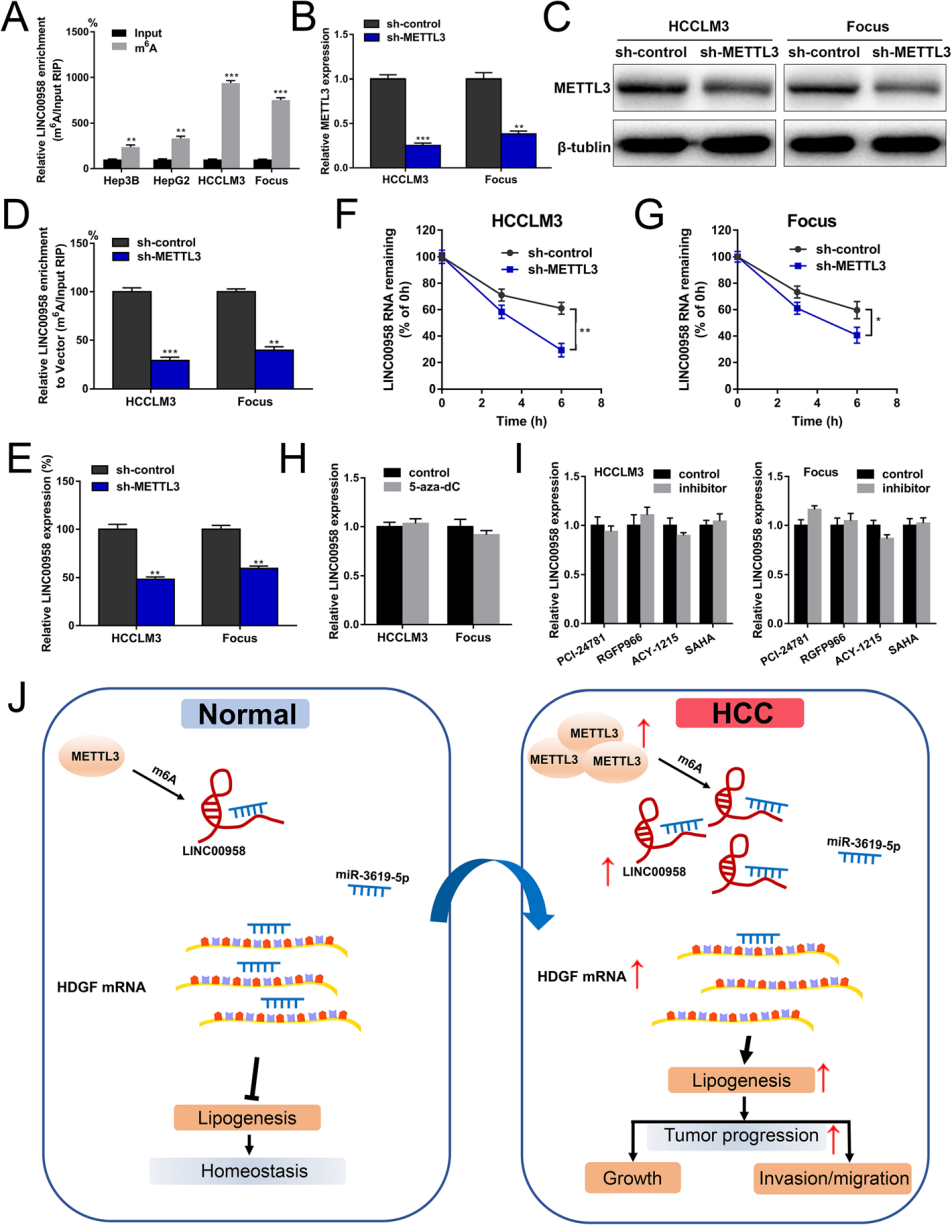
m^6^A modification is associated with LINC00958 upregulation in HCC cells. **a** m^6^A RIP-qPCR analysis showed that m^6^A was highly enriched within LINC00958 in HCC cells. **b** RT-qPCR was performed to verify the knockdown efficiency of METTL3 in HCCLM3 and Focus cells. **c** Western blotting was used to confirm the knockdown efficiency of METTL3 in HCCLM3 and Focus cells. **d** The m^6^A level of LINC00958 was examined in HCCLM3 and Focus cells with METTL3 knockdown. **e** The expression level of LINC00958 was assessed in HCCLM3 and Focus cells with METTL3 knockdown. **f** HCCLM3 cells with METTL3 knockdown were treated with actinomycin D for the indicated time points, and the expression level of LINC00958 was examined using RT-qPCR. **g** Focus cells with METTL3 knockdown were treated with actinomycin D for the indicated time points, and the expression level of LINC00958 was examined using RT-qPCR. **h** HCCLM3 and Focus cells were treated with or without 5-aza-dC, and LINC00958 expression level was examined using RT-qPCR. **i** HCCLM3 and Focus cells were treated with specific inhibitors of HDAC1 (PCI-24781), HDAC3 (RGFP966), HDAC6 (ACY-1215), and broad-spectrum HDAC inhibitor (SAHA). RT-qPCR was performed to examine the expression levels of LINC00958. **j** Schematic diagram demonstrating the molecular mechanisms underlying LINC00958 in HCC

Recent advancements in tumor epigenetic regulation have shed light on the involvement of m^6^A modification in lncRNA [26, 27]. We then wondered whether m^6^A was associated with LINC00958 upregulation in HCC. According to the results from an online bioinformatics database m6Avar [28], we found four RRACU m^6^A sequence motifs in the exon region (at ch11:13001568, 13002361, 13002410, and 13011005). m^6^A RIP-qPCR analysis showed that m^6^A was highly enriched within LINC00958 in Hep3B, HepG2, HCCLM3, and Focus cells (Fig. 7a). METTL3 is a crucial m^6^A methyltransferase and has been reported to be involved in HCC development [29]. RT-qPCR results indicated that METTL3 expression level was positively correlated with the level of LINC00958 in 50 HCC tissues (Additional file 12: Figure S7A). As indicated in Additional file 13: Table S3 and Additional file 12: Figure S7B, high METTL3 expression was associated with tumor differentiation (*P* = 0.002), tumor size (*P* = 0.018), microvascular invasion (*P* = 0.023), TNM stage (*P* = 0.001), and unfavorable overall survival. Patients in the LINC00958^high^METTL3^high^ group had the worst survival outcomes compared with other groups, indicating its prognostic value in HCC (Additional file 12: Figure S7C).

To explore the effects of METTL3 on LINC00958 upregulation in HCC, we knocked down the expression of METTL3 using lentivirus in HCCLM3 and Focus cells. As shown in Fig. 7b, c, RT-qPCR and western blotting assays verified the knockdown efficiency. Compared to the control group, the m^6^A level of LINC00958 was lower in METTL3-silenced HCC cells (Fig. 7d). We found that METTL3 downregulation was associated with decreased LINC00958 expression level (Fig. 7e). We then treated HCC cells with actinomycin D to block transcription and found that METTL3 knockdown significantly decreased the half-life of LINC00958 in HCCLM3 and Focus cells (Fig. 7f, g). These data suggested that METTL3-mediated m^6^A is associated with the upregulation of LINC00958 in HCC, probably by regulating the stability of its transcript. In addition, we investigated whether DNA methylation or histone modification could affect LINC00958 in HCC, whereas no significant results were observed (Fig. 7h, i). A graphic illustration of the tumor-promoting role of LINC00958 in HCC is depicted in Fig. 7j.

### Characteristics of PLGA-PEG(si-LINC00958) NPs

**Fig. 8:**
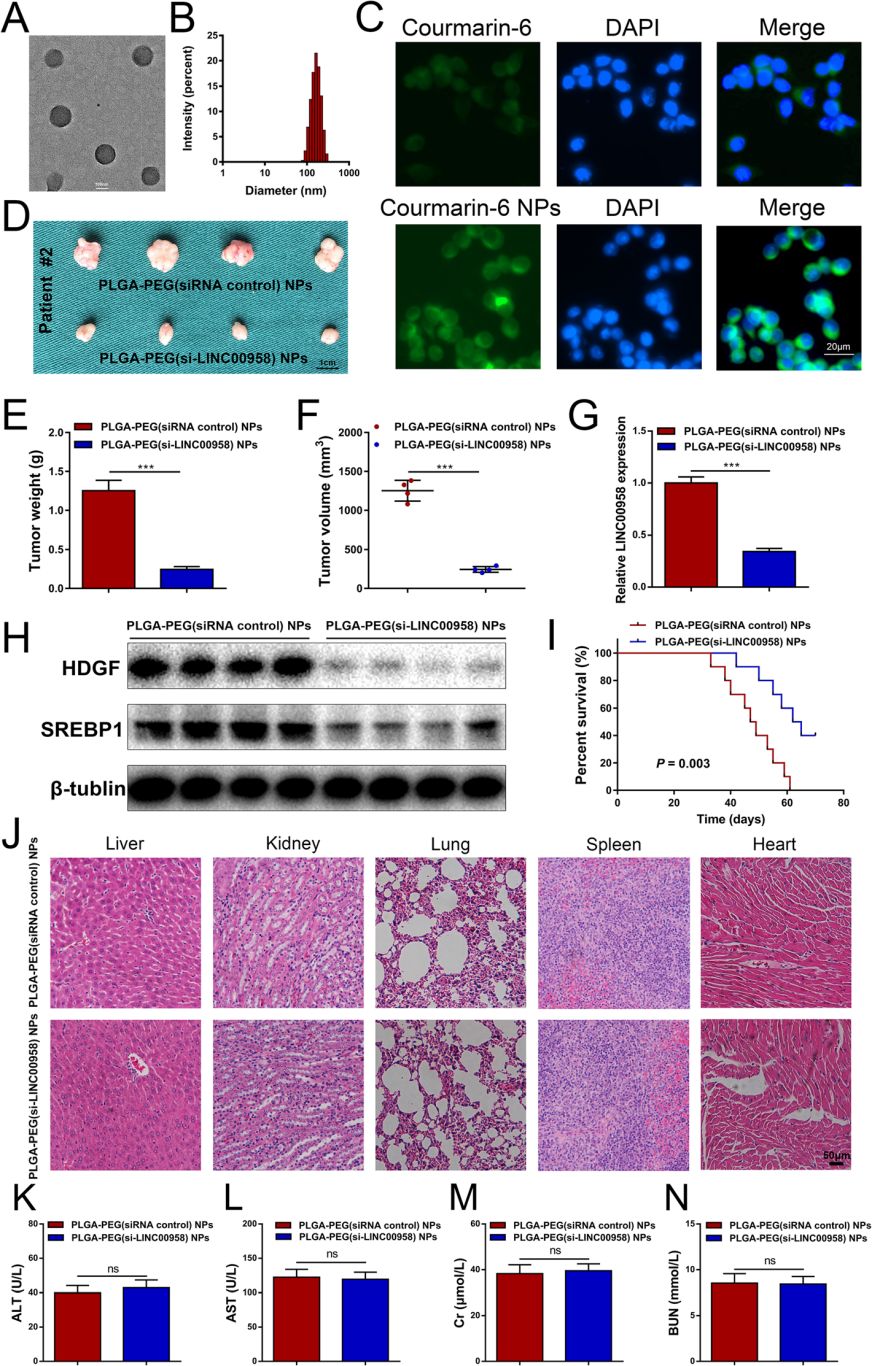
Characterization and therapeutic efficacy of PLGA-PEG(si-LINC00958) NPs in HCC PDX model. **a** Representative TEM image of PLGA-PEG(si-LINC00958) NPs. **b** The size distribution profile of PLGA-PEG(si-LINC00958) NPs. **c** Cellular uptake of NPs into HCCLM3 cells was evaluated by incubating HCCLM3 cells with Courmarin-6 labeled NPs. **d** HCC PDX model was used to demonstrate the therapeutic efficacy of PLGA-PEG(si-LINC00958) NPs via the tail vein injection. The harvested xenografted are shown. **e** Tumor weight was compared between PLGA-PEG(si-LINC00958) NPs and PLGA-PEG(siRNA control) NPs. **f** Tumor volume was compared between PLGA-PEG(si-LINC00958) NPs and PLGA-PEG(siRNA control) NPs. **g** RT-qPCR was performed to compare the expression level of LINC00958 between PLGA-PEG(si-LINC00958) NPs and PLGA-PEG(siRNA control) NPs. **h** Western blotting was performed to compare the expression level of LINC00958 between PLGA-PEG(si-LINC00958) NPs and PLGA-PEG(siRNA control) NPs. **i** Kaplan-Meier curves were plotted to compare the overall survival between the mice injected with PLGA-PEG(si-LINC00958) NPs and those injected with PLGA-PEG(siRNA control) NPs (*P* = 0.003). **j** Representative H&E staining of major organs including the liver, kidney, lung, spleen, and heart at the end of the experiment. **k** Blood ALT levels between the mice injected with PLGA-PEG(si-LINC00958) NPs and those injected with PLGA-PEG(siRNA control) NPs. **l** Blood AST levels between the mice injected with PLGA-PEG(si-LINC00958) NPs and those injected with PLGA-PEG(siRNA control) NPs. **m** Blood Cr levels between the mice injected with PLGA-PEG(si-LINC00958) NPs and those injected with PLGA-PEG(siRNA control) NPs. **n** BUN levels between the mice injected with PLGA-PEG(si-LINC00958) NPs and those injected with PLGA-PEG(siRNA control) NPs. ***P < 0.001

To investigate the potential utility of LINC00958 as a therapeutic target for HCC, we developed a novel PLGA-based nanoplatform encapsulating si-LINC00958. The double emulsion solvent diffusion method was used to prepare the PEGylated PLGA NPs loaded with si-LINC00958 and spermidine, named as PLGA-PEG(si-LINC00958) NPs hereafter. PEGylation of PLGA improves the stability of NPs in physiological environment by decreasing their interactions with serum proteins [30]. Spermidine can neutralize the charge of the anionic siRNA, turning it less hydrophilic and more likely to be encapsulated into hydrophobic PLGA [16]. As shown in representative TEM image (Fig. 8a), PLGA-PEG(si-LINC00958) NPs were spherical in shape and presented narrow size distributions. DLS were used to measure their size and zeta potential. The average diameter of PLGA-PEG(si-LINC00958) NPs was 170.49 ± 4.45 nm, with a PDI of 0.15 ± 0.01 (Fig. 8b and Additional file 14: Figure S8A). The zeta potential was − 4.85 ± 0.02 mV (Additional file 14: Figure S8B). A negative surface charge has been previously reported to be optimal to achieve a long-lasting in vivo circulation time [31]. The encapsulation efficiency of the PLGA-PEG(si-LINC00958) NPs was 40.8 ± 1.1%.

We then evaluated the in vitro release behavior of si-LINC00958 from PLGA-PEG(si-LINC00958) NPs (Additional file 14: Figure S8C). In the first 24 h, the cumulative release amount of siRNA from free si-LINC00958 and PLGA-PEG(si-LINC00958) NPs was 80.3% and 42.7%, respectively. As time went by, the si-LINC00958 entrapped in NPs was gradually released, and the sustained release continued for approximately 1 week. The data suggested that PLGA-PEG(si-LINC00958) NPs provided controlled release.

The cellular uptake of NPs into HCCLM3 cells was examined by incubating the cells with Courmarin-6 labeled NPs. Fluorescence microscopy revealed that Courmarin-6 NPs exhibited strong fluorescent signal inside the cells compared to the control (Fig. 8c), indicating that NPs increased the cellular drug uptake.

Then, we evaluated the targeting properties of NPs in vivo. Free 1,1′-dioctadecyl-3,3,3′,3′-tetramethylindotricarbocyanine iodide (DiR) or DiR-NP was intravenously injected via the tail vein. The fluorescent intensity of the DiR-NP group in tumor was significantly stronger than that of the free DiR group (Additional file 15: Figure S9A-B). The data demonstrated the in vivo tumor-targeting capacity of the NPs.

As shown in Additional file 14: Figure S8D, we verified the knockdown efficiency of PLGA-PEG(si-LINC00958) NPs in HCCLM3 cells. We then performed CCK-8 assays to assess the in vitro antitumor capability of PLGA-PEG(si-LINC00958) NPs. The results showed that PLGA-PEG(si-LINC00958) NPs could decrease the proliferative ability of HCC cells (Additional file 14: Figure S8E).

### Therapeutic efficacy and toxicity evaluation of systemic injection of PLGA-PEG(si-LINC00958) NPs in a PDX model of HCC

We treated the HCC PDX model by injecting PLGA-PEG(siRNA control) NPs or PLGA-PEG(si-LINC00958) NPs via the tail vein. Tumor growth was significantly inhibited following treatment with PLGA-PEG(si-LINC00958) NPs compared with PLGA-PEG(siRNA control) NPs (Fig. 8d). The xenograft tumor weight and volume were markedly reduced in mice injected with PLGA-PEG(si-LINC00958) NPs (Fig. 8e, f). RT-qPCR results confirmed the consistent knockdown of LINC00958 in xenografts derived from mice treated with PLGA-PEG(si-LINC00958) NPs (Fig. 8g). Downregulated expression levels of HDGF and SREBP1 in xenograft tumors were verified by western blotting (Fig. 8h). Survival analysis showed that systemic administration of PLGA-PEG(si-LINC00958) NPs remarkably prolonged the mouse overall survival (Fig. 8i).

We evaluated systemic toxicity by H&E staining and showed that intravenous administration of PLGA-PEG(si-LINC00958) NPs exhibited no significant toxicity to major organs including the liver, kidney, lung, spleen, and heart (Fig. 8j). In addition, we performed blood index analyses of ALT, AST, Cr, and BUN and confirmed the absence of significant hepatotoxicity and renotoxicity (Fig. 8k–n).

## Discussion

LncRNAs have been established as crucial regulators in pathogenesis, especially in malignancies [32]. In the present study, we used TCGA data to determine a landscape of differentially expressed lncRNAs in HCC, which revealed a significant upregulation of LINC00958 in HCC. We then confirmed that LINC00958 was highly expressed in HCC by RT-qPCR and FISH assays. High LINC00958 level was correlated with multiple malignant clinicopathological characteristics and was an independent predictor for unfavorable survival outcome. By loss-of-function and gain-of-function experiments, we demonstrated that LINC00958 promoted the proliferation, migration, and invasion of HCC in vitro. PDX models have emerged as invaluable preclinical models for cancer research [33]. We adopted PDX models and verified the tumor-promoting role of LINC00958 in vivo. Sequestration of miRNAs is the most frequently reported mechanism by which lncRNAs exert their regulatory function. Given the cytoplastic distribution of LINC00958 in HCC, we wondered whether LINC00958 could serve as a miRNA sponge. We screened six miRNAs overlapped by two different bioinformatics databases and verified the binding between LINC00958 and miR-3619-5p using RIP, dual luciferase reporter, RNA pull-down, and FISH assays. Further functional experiments showed that LINC00958 sponged miR-3619-5p to promote HCC progression. Previous studies indicated that miR-3619-5p inhibits cell proliferation and migration in HCC [34]. miR-3619-5p is involved in LINC00202-mediated retinoblastoma progression through targeting the expression of an oncogene RIN1 [35]. miR-3619-5p has also been demonstrated to exert a tumor-suppressive role in several types of malignancies, including bladder cancer [36], lung cancer [37], prostate cancer [38], and cutaneous squamous cell carcinoma [39].

To investigate the target gene of the LINC00958/miR-3619-5p pathway in HCC, we combined four bioinformatics algorithms and RNA sequencing results and found that HDGF was the downstream effector of the LINC00958/miR-3619-5p axis. HDGF has been established as an oncogene that facilitates the progression of HCC [40, 41]. One recent study suggested that HDGF could affect lipid metabolism via SREBP1 in HCC [25]. Our results revealed that LINC00958 facilitated lipogenesis via the miR-3619-5p/HDGF pathway. LINC00958 increased cellular cholesterol and triglyceride levels and contributed to lipid droplet formation. Key enzymes in lipogenesis, including SREBP1, FASN, SCD1, and ACC1, were also affected by LINC00958. As one of the hallmarks of cancer, metabolic alteration plays an indispensable role in cancer. However, only a few studies focused on the involvement of lncRNAs in HCC lipid metabolic reprogramming. Our data have provided novel insights into the lipogenesis-modulating role of LINC00958 in HCC.

Recent years have witnessed remarkable advancements of m^6^A modification in regulating all stages of the RNA life cycle. The deposition of m^6^A is encoded by “writers” that catalyze m^6^A formation (such as METTL3, METTL14, and WTAP), “erasers” that selectively remove the m^6^A code (such as FTO and ALKBH5), and “readers” that decode m^6^A methylation (such as YTH domain proteins and IGF2BP) [42]. m^6^A has been demonstrated to affect the targeted mRNA or miRNA and participate in the progression of various cancers [43]. However, studies on m^6^A modification in lncRNAs are scarce in the field of cancer. Recently, Wu et al. demonstrated that m^6^A modification upregulates lncRNA RP11 by increasing its nuclear accumulation [27]. m^6^A was suggested to be highly enriched on lncRNA FAM225A and can increase its RNA stability [26]. Herein, we revealed that m^6^A methylation was enriched within LINC00958 in HCC cells using both in silico data and m^6^A RIP experiment. Moreover, METTL3 regulated the m^6^A modification in LINC00958, thus affecting its RNA stability. These results suggested that elevation of LINC00958 in HCC may be attributed to the m^6^A modification.

Targeting delivery of siRNA using NPs has been recognized as practical and promising for cancer nanotherapy. Liposomes and viral vectors have been implicated to be potential vehicles for siRNA delivery, but they may induce toxicity and cannot maintain sustained release of siRNAs [16]. Approved by the Food and Drug Administration, PLGA is biodegradable and non-toxic and provides high stability, prolonged blood circulation time, and sustained release profile [44]. PLGA has gained substantial attention among the various polymers developed for formulation of nanoplatform and has been used for siRNA delivery. Byeon et al. used PLGA-based NPs incorporating FAK siRNA for overcoming chemoresistance in ovarian cancer [45]. PLGA NPs loaded with siRNA against osteopontin have been demonstrated to be effective for mammary carcinoma systemic treatment [46]. PEGylated NPs are regarded as “stealth NPs” and characterized by increased circulation time in vivo and tumor uptake. The surface shielding with PEG avoids plasma protein adsorption, protects NPs from the immune recognition, and increases bioavailability [18].

In this study, we developed and characterized a PEGylated PLGA nanoplatform loaded with LINC00958 siRNA for HCC therapy. PLGA-based nanosystem ensured the controlled release of si-LINC00958 and protected it from premature degradation. According to the results from cellular uptake experiments, NPs exhibited enhanced uptake into the tumor cells, which may facilitate the accumulation of NPs in the tumor. Biodistribution of NPs by systemic administration showed accumulated NPs in the xenograft tumor sites as well as the liver. The enhanced permeability and retention (EPR) effect is based on the leaky vasculature and poor lymphatic drainage present in the tumor. NPs > 100 nm can avoid being engulfed by the mononuclear phagocyte system and excreted by the kidney, while NPs < 400 nm preferentially accumulate in tumor sites and exhibit an optimal EPR effect [47]. Taking advantage of the EPR effect, PLGA-PEG(si-LINC00958) NPs achieved high concentration in HCC xenografts in vivo. Since the liver is the primary organ responsible for drug biotransformation, many NP-based drug delivery systems present substantial amounts of NPs in the liver [48, 49]. The results from PDX models demonstrated that this nanodrug system prominently reduced tumor burden. Compared with the control group, hampered tumor growth was observed in the PLGA-PEG(si-LINC00958) NP group. In addition, the results from H&E histopathological analysis and blood biochemical examination confirmed no significant toxic side effects.

## Conclusions

In summary, we comprehensively investigated the functional roles, molecular mechanisms, and clinical applications of LINC00958 in HCC. Our results revealed that LINC00958 was upregulated in HCC cell lines and tissues. High LINC00958 expression level was an independent prognostic factor for overall survival in HCC patients. We showed that LINC00958 promoted HCC cell proliferation, migration, invasion, and lipogenesis through the miR-3619-5p/HDGF axis. Moreover, PDX models were employed to confirm the effects of LINC00958 on HCC growth in vivo. We demonstrated that m^6^A modification was responsible for the upregulation of LINC00958 in HCC. For potential clinical application, we developed a novel nanoplatform encapsulating LINC00958 siRNA for HCC systemic treatment. Our study revealed that LINC00958 plays a crucial part in HCC lipogenesis and progression and highlighted its value as a prognostic predictor and nanotherapeutic candidate in HCC.

## Supplementary information

Additional file 1: Supplementary Methods

Additional file 2: **Figure S1.** LINC00958 is highly expressed in HCC and associated with overall survival. (A) The profile of differentially expressed lncRNAs in HCC was established based on TCGA data. The expression levels of 441 lncRNAs were found significantly altered in HCC tumor samples. (B) starBase Pan-Cancer Analysis Platform was used to examine the expression level of LINC00958 in liver cancer samples and normal samples (*P* = 0.0024). (C) Kaplan-Meier survival curves were plotted using starBase data to compare the overall survival between liver cancer patients with low LINC00958 level and those with high LINC00958 level (*P* = 0.014).

Additional file 3: **Table S1.** The correlation between clinicopathological characteristics and LINC00958 expression level in 80 hepatocellular carcinoma patients.

Additional file 4: **Table S2.** Univariate and multivariable analysis of overall survival after surgery.

Additional file 5: **Figure S2.** LINC00958 overexpression facilitates HCC proliferation, migration, and invasion in vitro. (A) Lentiviruses were used to upregulate the expression of LINC00958 in Hep3B and HepG2 cells. (B) CCK-8 assays were performed to assess the cell proliferation in LINC00958-overexpressed Hep3B and HepG2 cells. (C) Colony formation assays showed the clone numbers in HCC cells with LINC00958 overexpression. (D) EdU assays were performed to assess the proliferative ability of Hep3B and HepG2 cells with LINC00958 overexpression. (E) Transwell assays were conducted to examine the effects of LINC00958 overexpression on HCC cell migration and invasion. *P < 0.05, **P < 0.01, ***P < 0.001.

Additional file 6: Supplementary Material 1. miRNAs with complementary sequences to LINC00958 predicted by starBase and miRDB.

Additional file 7: **Figure S3.** LINC00958 exerts its tumor-promoting effects via miR-3619-5p. (A) The efficiency of miR-3619-5p mimics in LINC00958-overexpressed Hep3B cells was confirmed using RT-qPCR. (B) CCK-8 assays were conducted to evaluate the effects of miR-3619-5p overexpression on the proliferative ability of LINC00958-overexpressed Hep3B cells. The data are shown as the mean ± SEM. *P < 0.05, **P < 0.01 vs. the NC group. (C) EdU assays were performed to analyze the effects of miR-3619-5p overexpression on LINC00958-overexpressed Hep3B cells. The data are shown as the mean ± SEM. **P < 0.01 vs. the NC group. (D) Transwell assays were conducted to evaluate the effects of miR-3619-5p overexpression on the migration and invasion of LINC00958-overexpressed Hep3B cells. The data are shown as the mean ± SEM. ***P < 0.001 vs. the NC group.

Additional file 8: Supplementary Material 2. Targets of miR-3619-5p predicted by PicTar, TargetScan, miRDB, and RNA22.

Additional file 9: **Figure S4.** The LINC00958/miR-3619-5p axis affects HCC progression through HDGF. (A) The efficiency of HDGF overexpression in LINC00958-silenced HCCLM3 cells was verified using RT-qPCR. (B) Western blotting was performed to validate the efficiency of HDGF overexpression in LINC00958-silenced HCCLM3 cells. (C) CCK-8 assays were conducted to evaluate the effects of HDGF overexpression on the proliferative ability of LINC00958-silenced HCCLM3 cells. The data are shown as the mean ± SEM. **P < 0.01 vs. the sh-NC group. (D) EdU assays were performed to analyze the effects of HDGF overexpression on Hep3B cells with LINC00958 knockdown. The data are shown as the mean ± SEM. **P < 0.01 vs. the sh-NC group. (E) Transwell assays were conducted to evaluate the effects of HDGF overexpression on the migration and invasion of LINC00958-silenced Hep3B cells. The data are shown as the mean ± SEM. **P < 0.01, ***P < 0.001 vs. the sh-NC group.

Additional file 10: **Figure S5.** LINC00958 promotes lipogenesis through miR-3619-5p. (A) Effects of miR-3619-5p overexpression on cholesterol level in LINC00958-overexpressed Hep3B cells. The data are shown as the mean ± SEM. ***P < 0.001 vs. the NC group. (B) Effects of miR-3619-5p overexpression on triglyceride level in LINC00958-overexpressed Hep3B cells. The data are shown as the mean ± SEM. ***P < 0.001 vs. the NC group. (C) RT-qPCR assays were used to examine the effects of miR-3619-5p overexpression on SREBP1 level in LINC00958-overexpressed Hep3B cells. The data are shown as the mean ± SEM. ***P < 0.001 vs. the NC group. (D) Western blotting was performed to investigate the effects of miR-3619-5p overexpression on SREBP1 level in LINC00958-overexpressed Hep3B cells. (E) Oil Red O staining showing the effects of miR-3619-5p overexpression on lipid droplet formation in LINC00958-overexpressed Hep3B cells.

Additional file 11: **Figure S6.** The LINC00958/miR-3619-5p pathway modulates lipogenesis by targeting HDGF. (A) Effects of HDGF overexpression on cholesterol level in LINC00958-silenced HCCLM3 cells. The data are shown as the mean ± SEM. ***P < 0.001 vs. the sh-NC group. (B) Effects of HDGF overexpression on triglyceride level in LINC00958-silenced HCCLM3 cells. The data are shown as the mean ± SEM. ***P < 0.001 vs. the sh-NC group. (C) RT-qPCR assays were used to examine the effects of HDGF overexpression on SREBP1 level in LINC00958-silenced HCCLM3 cells. The data are shown as the mean ± SEM. ***P < 0.001 vs. the sh-NC group. (D) Western blotting was performed to investigate the effects of HDGF overexpression on SREBP1 level in LINC00958-silenced HCCLM3 cells. (E) Oil Red O staining showing the effects of HDGF overexpression on lipid droplet formation in LINC00958-silenced HCCLM3 cells.

Additional file 12: **Figure S7.** The correlation between METTL3 expression and LINC00958 levels in HCC patient samples and its clinical impact. (A) Correlation analysis showing a positive correlation between LINC00958 and METTL3 expression (*P* < 0.001). (B) Kaplan-Meier survival curves showing the effect of METTL3 on overall survival (*P* = 0.001). (C) Kaplan-Meier survival curves showing the effect of the combination of LINC00958 and METTL3 on overall survival (*P* < 0.001).

Additional file 13: **Table S3.** The correlation between clinicopathological characteristics and METTL3 expression level in 50 hepatocellular carcinoma patients.

Additional file 14: **Figure S8.** Characterization, release behavior, and anti-proliferative capability of NPs in vitro. (A) DLS measurements of the size and PDI of PLGA-PEG(si-LINC00958) NPs for 1 week. (B) Zeta potential of PLGA-PEG(si-LINC00958) NPs. (C) In vitro release of LINC00958 siRNA from PLGA-PEG(si-LINC00958) NPs at 37 °C. (D) The knockdown efficiency of PLGA-PEG(si-LINC00958) NPs in HCCLM3 cells was evaluated using RT-qPCR. (E) CCK-8 assays were used to examine the in vitro anti-tumor capability of PLGA-PEG(si-LINC00958) NPs. **P < 0.01, ***P < 0.001.

Additional file 15: **Figure S9.** Biodistribution of NPs by systemic injection. (A) In vivo dynamic fluorescence imaging after injecting free DiR or DiR-NP via tail vein. The xenograft tumor sites are indicated by red arrows. (B) Fluorescence intensities of the xenograft tumor sites were quantified using IVIS Lumina XRMS In Vivo Imaging System. ***P < 0.001.

## Authors’ information

Authors’ information has been mentioned in the cover page.

## Abbreviations

ALT: Alanine transaminase
AST: Aspartate transaminase
BUN: Blood urea nitrogen
CI: Confidence interval
Cr: Creatinine
DiR: 1,1′-Dioctadecyl-3,3,3′,3′-tetramethylindotricarbocyanine iodide
DLS: Dynamic light scattering
EPR: Enhanced permeability and retention
FISH: Fluorescence in situ hybridization
H&E: Hematoxylin-eosin
HCC: Hepatocellular carcinoma
HDGF: Hepatoma-derived growth factor
HR: Hazard ratio
lncRNA: Long non-coding RNAs
NC: Negative control
NP: Nanoparticle
PDI: Polydispersity index
PDX: Patient-derived xenograft
PEG: Poly(ethylene glycol)
PLGA: Poly(lactic acid/glycolic)
RIP: RNA immunoprecipitation
shRNA: Small hairpin RNA
siRNA: Small interfering RNAs
TEM: Transmission electron microscopy
