# Supplementary material for: M6A-mediated upregulation of LINC00958 increases lipogenesis and acts as a nanotherapeutic target in hepatocellular carcinoma
Source: J Hematol Oncol. 2020 Jan 8;13:5. doi: 10.1186/s13045-019-0839-x (PMC6951025; doi:10.1186/s13045-019-0839-x)
Supplement: Supplementary file 1 — Supplementary Methods [file 13045_2019_839_MOESM1_ESM.docx]

**Additional file 1: Supplementary Methods**

**Cell lines and cell culture**

The normal hepatic cell line QSG-7701 and HCC cell lines HepG2 and Hep3B were purchased from American Type Culture Collection (Rockville, MD, USA). Huh7 cell lines were obtained from the Cell Bank of the Chinese Academy of Sciences (Shanghai, China). MHCC-97H and HCCLM3 cell lines were acquired from the Liver Cancer Institute, Zhongshan Hospital, Fudan University (Shanghai, China). Focus cell line was obtained from the NHC Key Laboratory of Liver Transplantation (Nanjing, China). Cell lines were regularly examined to be free of mycoplasma contamination and used within 6 months after resuscitation of frozen aliquots. All cells were cultured in Dulbecco’s Modified Eagle’s Medium (DMEM; Gibco, Carlsbad, CA, USA) supplemented with 10% fetal bovine serum (FBS; Invitrogen, Carlsbad, CA, USA) and 1% penicillin/streptomycin (Gibco). Cells were incubated in a humidified atmosphere containing 5% CO_2_ at 37℃.

**RNA isolation and quantitative real-time polymerase chain reaction (RT-qPCR)**

Total RNA was extracted using TRIzol reagent (Invitrogen). Reverse transcription was performed using PrimeScript RT Reagent Kit (TaKaRa, Dalian, China) with Bulge-Loop miRNA-specific RT primers (RiboBio, Guangzhou, China) for miRNA or random primers for mRNA and lncRNA. We performed RT-qPCR by SYBR Premix Ex Taq (TaKaRa) on ABI 7900HT Real-time PCR System (Applied Biosystems, Foster City, CA, USA). The NE-PER Nuclear and Cytoplasmic Extraction Reagents (Thermo Fisher Scientific, Carlsbad, CA, USA) were adopted to isolate the nuclear and cytoplasmic fractions based on the protocol provided by the manufacturer. The primer sequences are showed as follows: LINC00958: forward: 5’-CCATTGAAGATACCACGCTGC-3’, reverse: 5’-GGTTGTTGCCCAGGGTAGTG-3’; HDGF: forward: 5’-CTCTTCCCTTACGAGGAATCCA-3’, reverse: 5’-CCTTGACAGTAGGGTTGTTCTC-3’ ; SREBP1: forward: 5’-GCTGCTGACCGACATCGAA-3’, reverse: 5’-CCAGCATAGGGTGGGTCAAA-3’; FASN: forward: 5’-AGTACACACCCAAGGCCAAG-3’, reverse: 5’-GGATACTTTCCCGTCGCATA-3’; SCD1: forward: 5’-GCAGGACGATATCTCTAGCT-3’, reverse: 5’-GTCTCCAACTTATCTCCTCCATTC-3’; ACC1: forward: 5’-GAGGGAAGGGAATTAGAA-3’, reverse: 5’-ATCACCCCAGGGAGATAC-3’; METTL3: forward: 5’-ATCCCCAAGGCTTCAACCAG-3’, reverse: 5’-GCGAGTGCCAGGAGATAGTC-3’; GAPDH: forward: 5′-AATCCCATCACCATCTTCC-3′; reverse: 5′-CATCACGCCACAGTTTCC-3′; β-actin: forward: 5’- TGACGTGGACATCCGCAAAG-3’, reverse: 5’-CTGGAAGGTGGACAGCGAGG-3’. The RT-qPCR primers for miRNAs and U6 were designed and synthesized by RiboBio.

**Cell proliferation assays**

Cell viability were evaluated by Cell Counting Kit-8 (CCK-8; Dojindo, Kumamoto, Japan), colony formation, and 5-ethynyl-2’-deoxyuridine (EdU) incorporation assays. For CCK-8 assay, a total of 1 × 10^3^ cells were seeded into 96-well plates and incubated overnight. Then, 10 μl of CCK-8 solution reagent was added to each well at the indicated time points (24 h, 48 h, 72 h, 96 h, and 120 h) and the cells were incubated in dark for 2 h. Subsequently, the absorbance at the wavelength of 450 nm was measured by a micro-plate reader (Bio-Tek Elx 800; Bio-Tek Instruments, Winooski, VT, USA). For colony formation assay, cells were seeded into 6-well plates at a density of 5 × 10^2^ cells/well. After the cells were cultured for two weeks, the colonies were fixed in 4% paraformaldehyde and stained with 1% crystal violet. For EdU assay, we used Cell-Light EdU Apollo 488 *In Vitro* Imaging Kit (RiboBio, Guangzhou, China) to detect the DNA synthesis of HCC cells. Briefly, a total of 5 × 10^4^ cells were cultured in 24-well plates. Subsequently, the cells were incubated with EdU solution for 2 h, reacted with 1 × Apollo reaction cocktail for 30 min, and stained with Hoechst 33342 for 30 min.

**Transwell assay**

The cell invasion and migration assays were performed using 24-well transwell chambers (Corning Inc., Corning, NY, USA). For cell invasion assay, the upper chambers were coated with Matrigel (BD Biosciences, San Diego, CA, USA). A total of 2 × 10^4^ cells were seeded into the upper chamber with 250 μl of serum-free medium. Then 500 μl of DMEM containing 10% FBS was added to the lower chambers. After incubation for 24 h, the invaded cells were fixed with 4% paraformaldehyde, stained with 1% crystal violet and photographed under a microscope. Cell migration assay was performed similarly without coating upper chambers with Matrigel.

**Luciferase reporter gene assays**

The luciferase activity was assessed using the Dual Luciferase Reporter Assay System (Promega, Massachusetts, WI, USA). Briefly, the wildtype or mutant sequences of LINC00958 or HDGF 3’-UTR were subcloned into pmirGLO vector. HCC cells were co-transfected with blank pmirGLO, LINC00958-WT, LINC00958-MUT, HDGF 3’-UTR-WT, or HDGF 3’-UTR-MUT as well as miR-3619-5p mimics or miR-control. After 48 h of transfection, cells were lysed and the luciferase activity was measured.

**Triglycerides and cholesterol assays**

We used the triglyceride assay kit and the cholesterol assay kit (Nanjing Jiancheng Bioengineering Institute, Nanjing, China) to determine the intracellular triglyceride and cholesterol levels with a micro-plate reader (Bio-Tek Instruments) according to the manufacturer's instructions.

**Western blotting**

HCC cells were harvested and lysed in RIPA buffer with PMSF (Beyotime, Shanghai, China). The BCA Protein Assay Kit (Beyotime) was used to determine the total protein concentration. Protein samples were separated by sodium dodecyl sulfate-polyacrylamide gel electrophoresis and transferred onto polyvinylidene difluoride membranes (Millipore). Then, these membranes were blocked with 5% non-fat milk, probed with primary antibodies at 4℃ overnight and incubated with the corresponding HRP-conjugated secondary antibody. Each specific blotting band was detected by enhanced chemilu­minescence reagents (Yeasen, Shanghai, China). The antibodies used for western blotting were list as follow: HDGF (ab128921, Abcam), SREBP1 (ab28481, Abcam), FASN (#3180, Cell Signaling Technology, Beverly, MA, USA), SCD1 (ab19862, Abcam), ACC1 (ab45174, Abcam), METTL3 (ab195352, Abcam), β-tublin (#2146, Cell Signaling Technology), HRP-linked anti-rabbit IgG (#7074, Cell Signaling Technology), and HRP-linked anti-mouse IgG (#7076, Cell Signaling Technology).

**Immunohistochemistry**

Xenografts were fixed in 4% paraformaldehyde and the paraffin-embedded tissues were cut into 4-μm sections. After blocking endogenous activity, sections were incubated with primary antibodies at 4°C overnight, followed by HRP-conjugated secondary antibody at 37°C for 1 h. Next, the sections were stained with HRP substrate DAB. The antibodies used for immunohistochemistry are shown as follows: HDGF (ab128921, Abcam), SREBP1 (ab28481, Abcam), and Ki67 (ab16667, Abcam).

**Actinomycin D assay**

To measure LINC00958 stability, 5 μg/ml of actinomycin D (Sigma-Aldrich, St. Louis, MO, USA) was added to HCCLM3 and Focus cells. After incubation for different time points, cells were harvested, and the relative RNA levels of LINC00958 were detected by RT-qPCR.

**5-aza-dC and HDAC inhibitor treatment**

To investigate the effect of DNA methylation on LINC00958 expression, HCCLM3 and Focus cells were exposed to 5 μM of 5-aza-dC (Sigma-Aldrich) for six days. The expression level of LINC00958 was then measured by RT-qPCR. For histone modification analysis, HCC cells were treated with specific inhibitors of HDAC1 (PCI-24781, 8 µM), HDAC3 (RGFP966, 1 µM), HDAC6 (ACY-1215, 4 µM), or broad-spectrum HDAC inhibitor (SAHA, 2 µM) for 24 h, and LINC00958 expression level was examined by RT-qPCR.

***In vitro* release of LINC00958 siRNA**

The release profile of siRNA from NPs was evaluated using a dialysis method. Briefly, the specimens were kept in a dialysis bag (GreenBird Inc., Shanghai, China) and dialyzed against PBS (pH = 7.4). The samples were incubated at 37°C under gentle shaking. At indicated time points, the concentration of siRNA was measured using UV spectrophotometry.

**Cellular uptake of NPs**

For cellular uptake assessment, the fluorescent probe Coumarin-6 (Sigma-Aldrich) was used to label the PLGA-PEG NPs. HCCLM3 cells were seeded in a 6-well plate and incubated overnight. Coumarin-6 NPs or free Coumarin-6 was added into the culture medium at a concentration of 2 μg/mL of Coumarin-6. DAPI was used to label the nuclei. Fluorescence microscope (EVOS FL Auto, Tokyo, Japan) was used to observe the cellular uptake of Coumarin-6.

**Biodistribution of NPs in tumor-bearing mice by systemic injection**

Lipophilic carbocyanine dye DiR (MaokangBio, Shanghai, China) was used to evaluate the *in vivo* tumor-targeting ability of the PLGA-PEG NPs. We construct xenograft mouse models by subcutaneously injecting 100 μL of cell suspension comprising 5 × 10^6^ HCCLM3 cells into the flank of nude mice. Free DiR or DiR-loaded NPs were intravenously injected via tail vein. Images were captured at indicated time points using IVIS Lumina XRMS *In Vivo* Imaging System (PerkinElmer, Waltham, MA, USA).
