# Supplementary figures and images for: M6A-mediated upregulation of LINC00958 increases lipogenesis and acts as a nanotherapeutic target in hepatocellular carcinoma
Source: J Hematol Oncol. 2020 Jan 8;13:5. doi: 10.1186/s13045-019-0839-x (PMC6951025; doi:10.1186/s13045-019-0839-x)

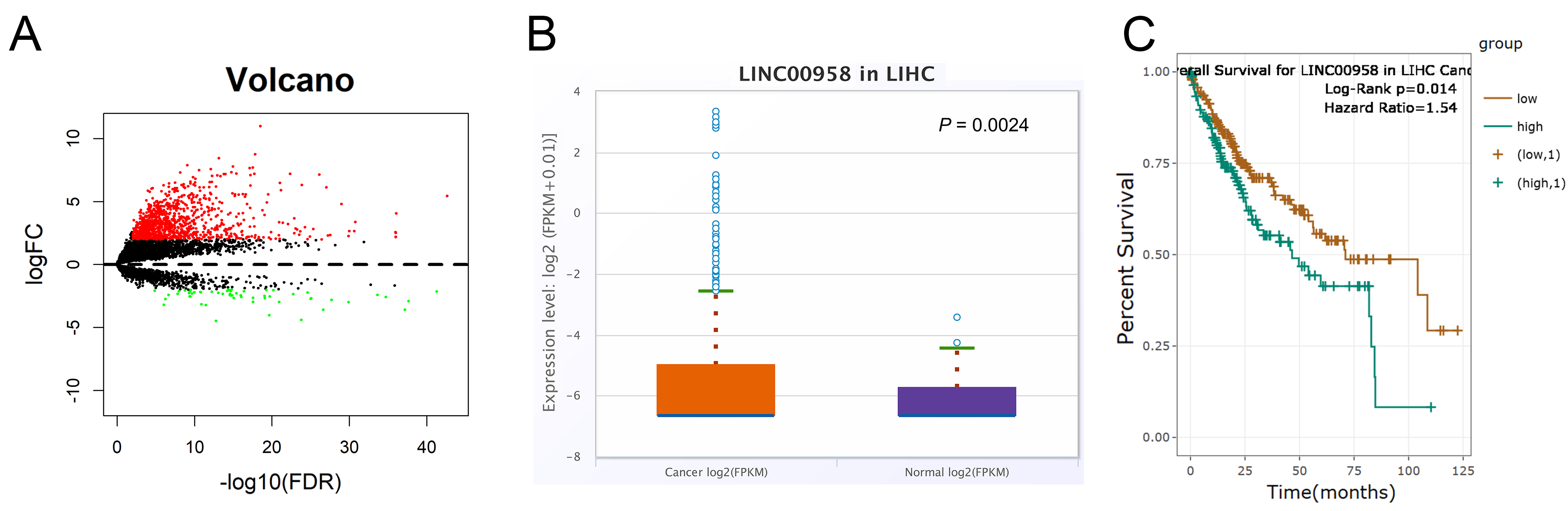

Supplement: Supplementary file 2 — Figure S1. LINC00958 is highly expressed in HCC and associated with overall survival. (A) The profile of differentially expressed lncRNAs in HCC was established based on TCGA data. The expression levels of 441 lncRNAs were found significantly altered in HCC tumor samples. (B) starBase Pan-Cancer Analysis Platform was used to examine the expression level of LINC00958 in liver cancer samples and normal samples (P = 0.0024). (C) Kaplan-Meier survival curves were plotted using starBase data to compare the overall survival between liver cancer patients with low LINC00958 level and those with high LINC00958 level (P = 0.014). [file 13045_2019_839_MOESM2_ESM.tif]

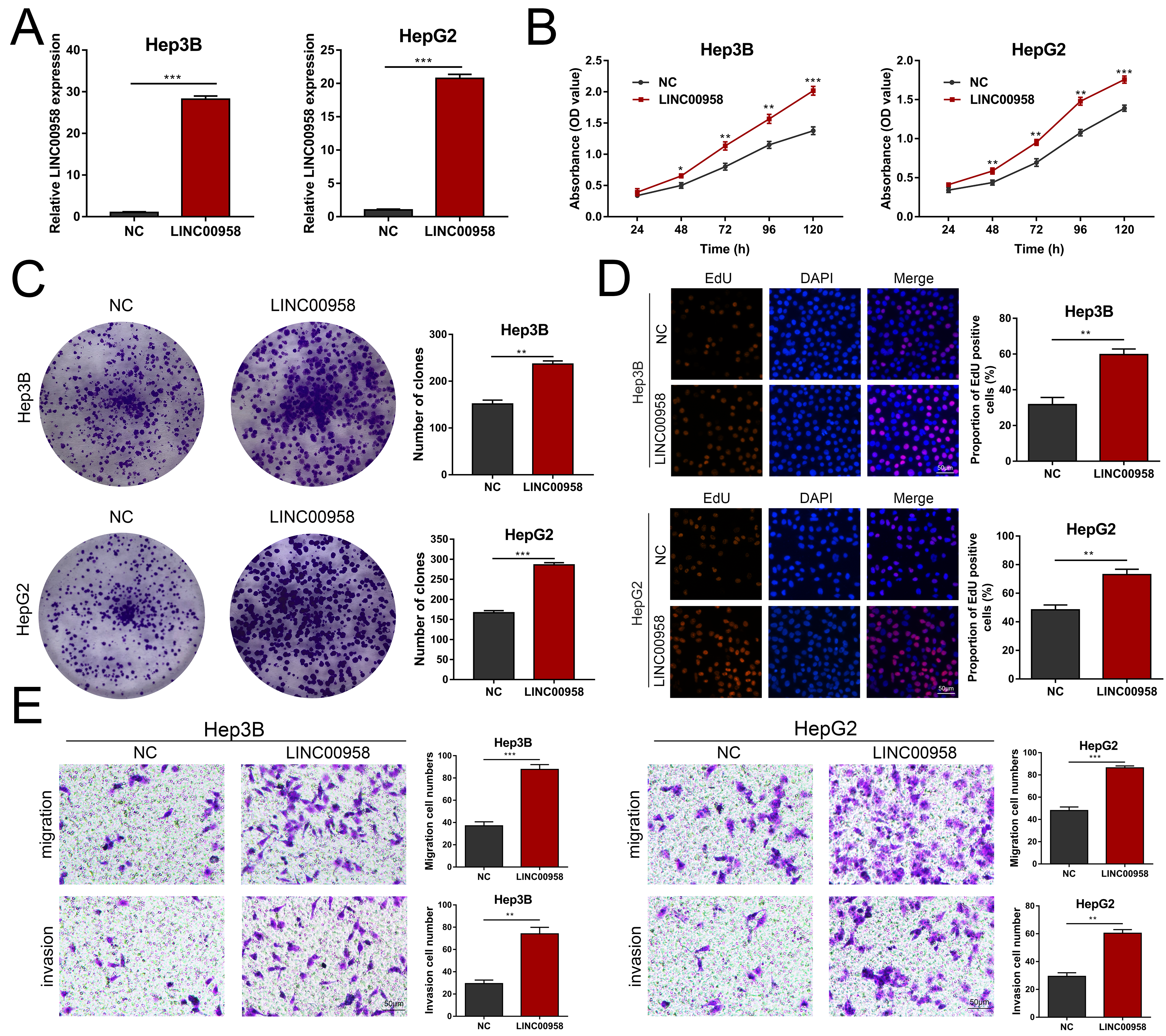

Supplement: Supplementary file 5 — Figure S2. LINC00958 overexpression facilitates HCC proliferation, migration, and invasion in vitro. (A) Lentiviruses were used to upregulate the expression of LINC00958 in Hep3B and HepG2 cells. (B) CCK-8 assays were performed to assess the cell proliferation in LINC00958-overexpressed Hep3B and HepG2 cells. (C) Colony formation assays showed the clone numbers in HCC cells with LINC00958 overexpression. (D) EdU assays were performed to assess the proliferative ability of Hep3B and HepG2 cells with LINC00958 overexpression. (E) Transwell assays were conducted to examine the effects of LINC00958 overexpression on HCC cell migration and invasion. *P < 0.05, **P < 0.01, ***P < 0.001. [file 13045_2019_839_MOESM5_ESM.tif]

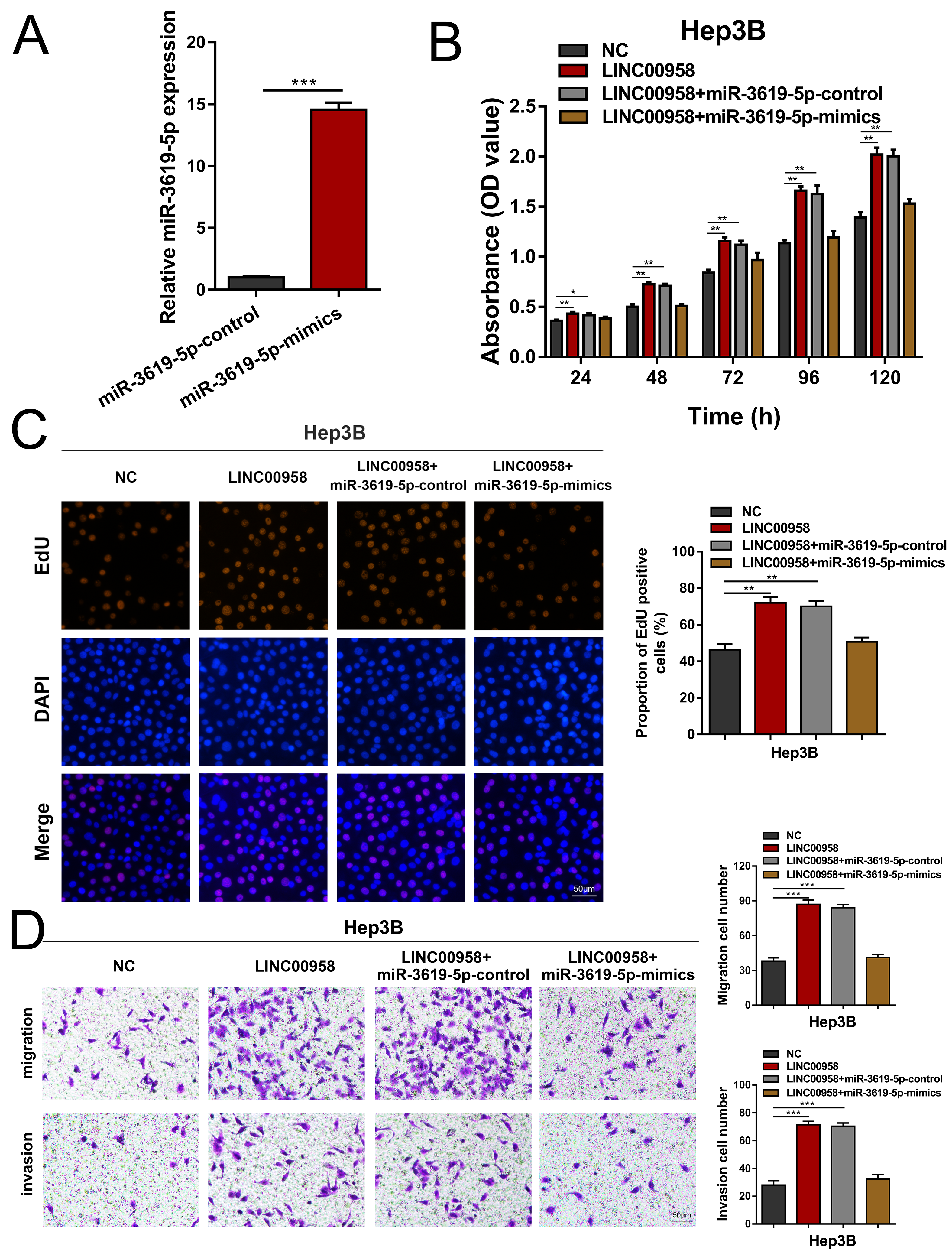

Supplement: Supplementary file 7 — Figure S3. LINC00958 exerts its tumor-promoting effects via miR-3619-5p. (A) The efficiency of miR-3619-5p mimics in LINC00958-overexpressed Hep3B cells was confirmed using RT-qPCR. (B) CCK-8 assays were conducted to evaluate the effects of miR-3619-5p overexpression on the proliferative ability of LINC00958-overexpressed Hep3B cells. The data are shown as the mean ± SEM. *P < 0.05, **P < 0.01 vs. the NC group. (C) EdU assays were performed to analyze the effects of miR-3619-5p overexpression on LINC00958-overexpressed Hep3B cells. The data are shown as the mean ± SEM. **P < 0.01 vs. the NC group. (D) Transwell assays were conducted to evaluate the effects of miR-3619-5p overexpression on the migration and invasion of LINC00958-overexpressed Hep3B cells. The data are shown as the mean ± SEM. ***P < 0.001 vs. the NC group. [file 13045_2019_839_MOESM7_ESM.tif]

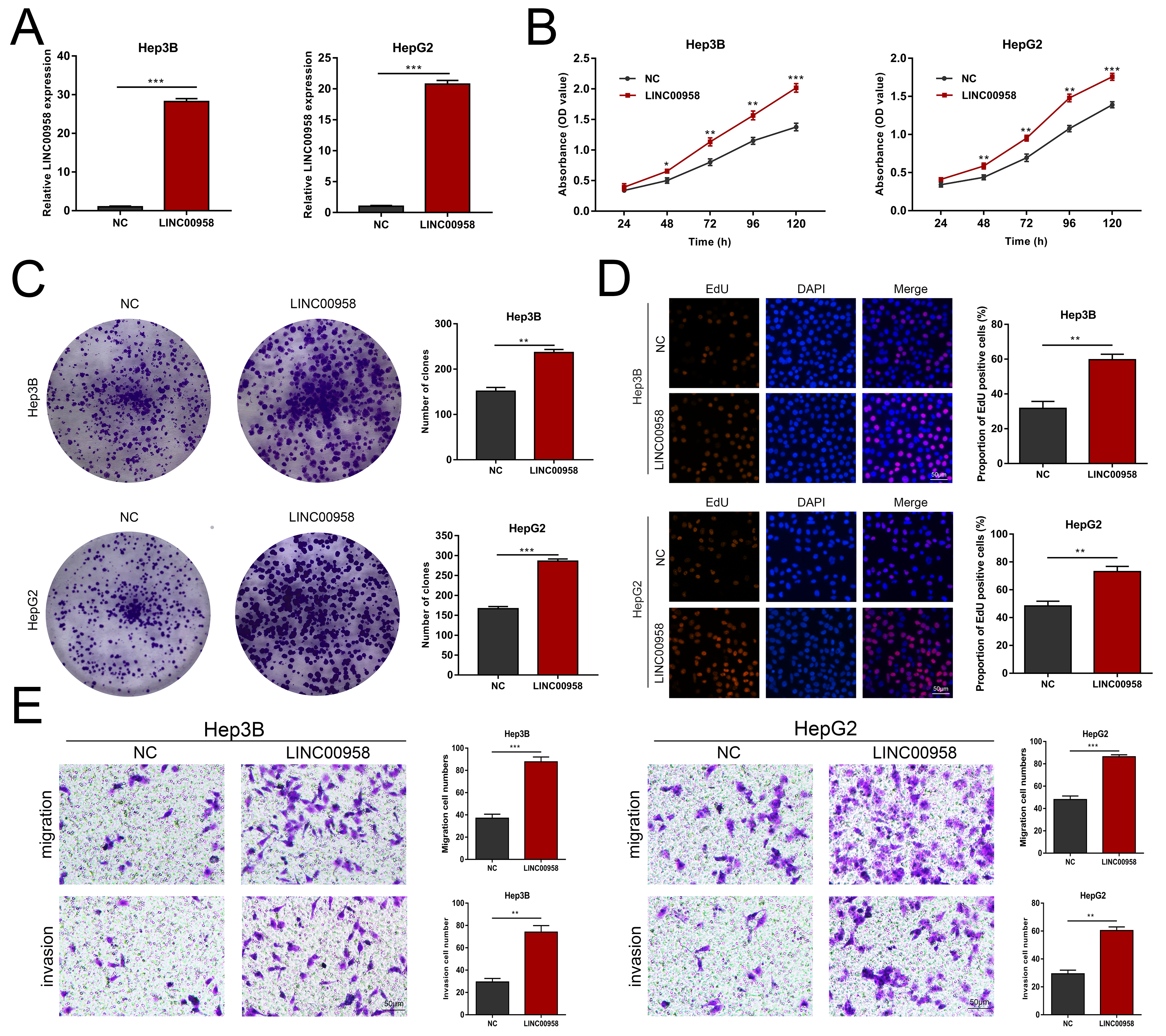

Supplement: Supplementary file 8 — Supplementary Material 2. Targets of miR-3619-5p predicted by PicTar, TargetScan, miRDB, and RNA22. [file 13045_2019_839_MOESM8_ESM.tif]

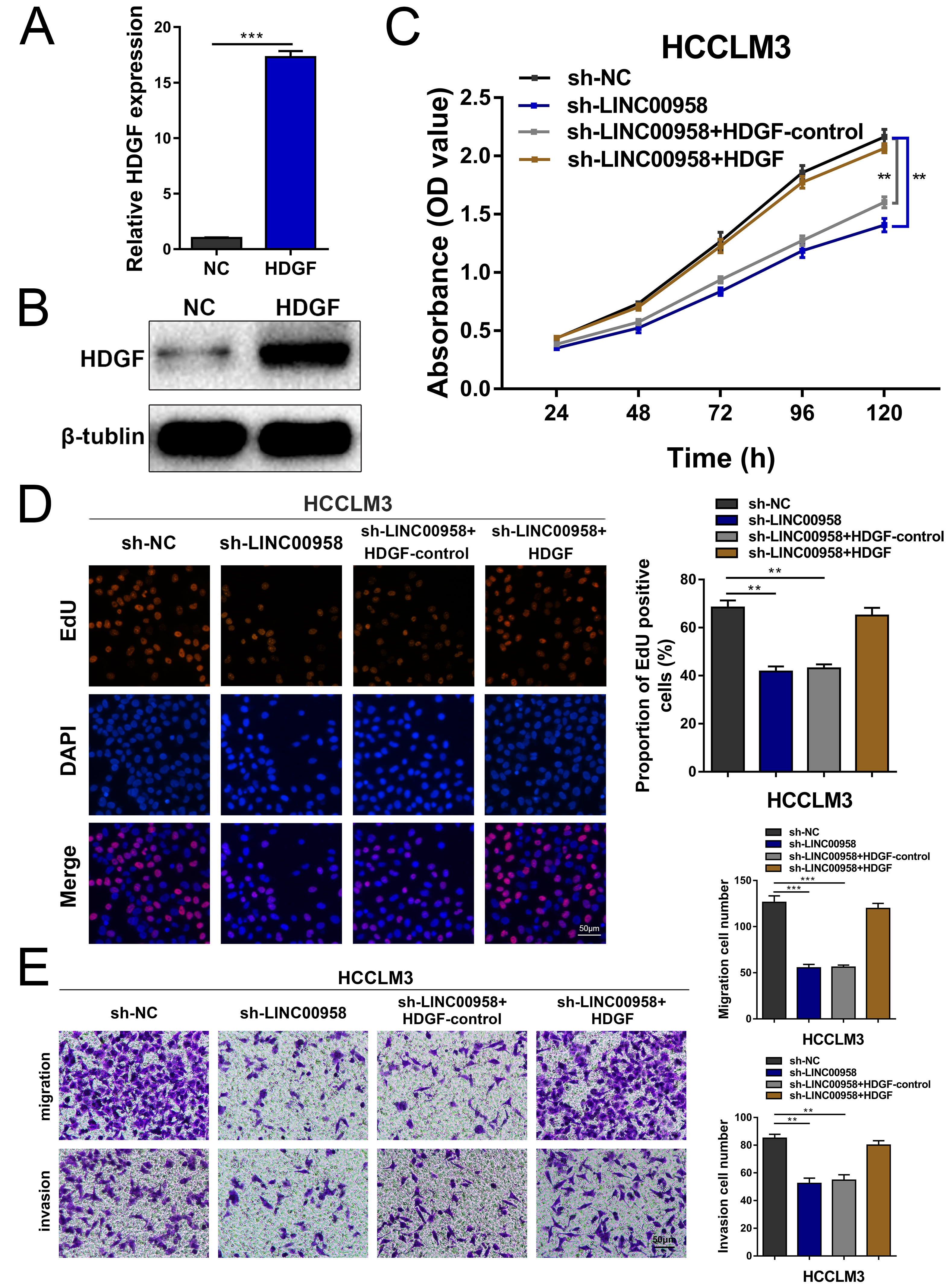

Supplement: Supplementary file 9 — Figure S4. The LINC00958/miR-3619-5p axis affects HCC progression through HDGF. (A) The efficiency of HDGF overexpression in LINC00958-silenced HCCLM3 cells was verified using RT-qPCR. (B) Western blotting was performed to validate the efficiency of HDGF overexpression in LINC00958-silenced HCCLM3 cells. (C) CCK-8 assays were conducted to evaluate the effects of HDGF overexpression on the proliferative ability of LINC00958-silenced HCCLM3 cells. The data are shown as the mean ± SEM. **P < 0.01 vs. the sh-NC group. (D) EdU assays were performed to analyze the effects of HDGF overexpression on Hep3B cells with LINC00958 knockdown. The data are shown as the mean ± SEM. **P < 0.01 vs. the sh-NC group. (E) Transwell assays were conducted to evaluate the effects of HDGF overexpression on the migration and invasion of LINC00958-silenced Hep3B cells. The data are shown as the mean ± SEM. **P < 0.01, ***P < 0.001 vs. the sh-NC group. [file 13045_2019_839_MOESM9_ESM.tif]

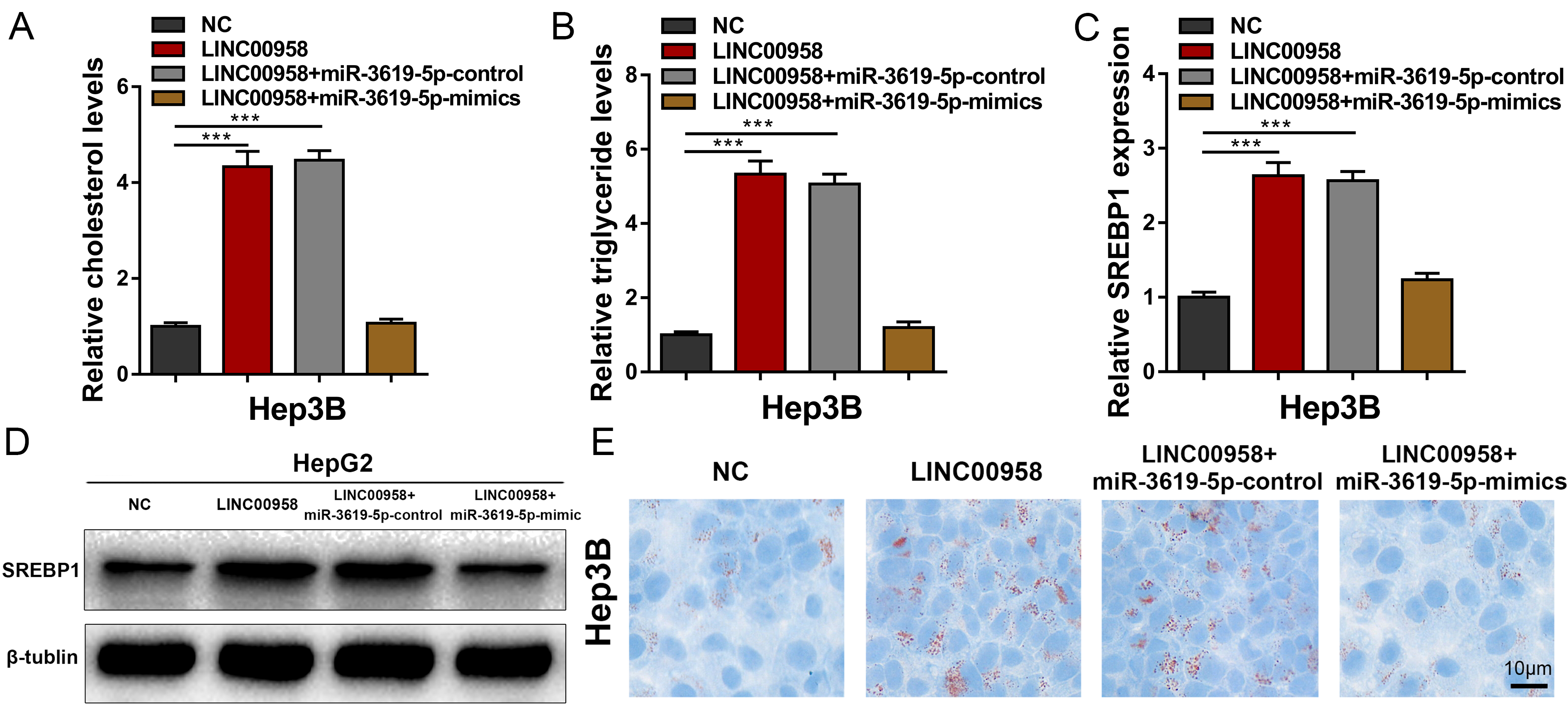

Supplement: Supplementary file 10 — Figure S5. LINC00958 promotes lipogenesis through miR-3619-5p. (A) Effects of miR-3619-5p overexpression on cholesterol level in LINC00958-overexpressed Hep3B cells. The data are shown as the mean ± SEM. ***P < 0.001 vs. the NC group. (B) Effects of miR-3619-5p overexpression on triglyceride level in LINC00958-overexpressed Hep3B cells. The data are shown as the mean ± SEM. ***P < 0.001 vs. the NC group. (C) RT-qPCR assays were used to examine the effects of miR-3619-5p overexpression on SREBP1 level in LINC00958-overexpressed Hep3B cells. The data are shown as the mean ± SEM. ***P < 0.001 vs. the NC group. (D) Western blotting was performed to investigate the effects of miR-3619-5p overexpression on SREBP1 level in LINC00958-overexpressed Hep3B cells. (E) Oil Red O staining showing the effects of miR-3619-5p overexpression on lipid droplet formation in LINC00958-overexpressed Hep3B cells. [file 13045_2019_839_MOESM10_ESM.tif]

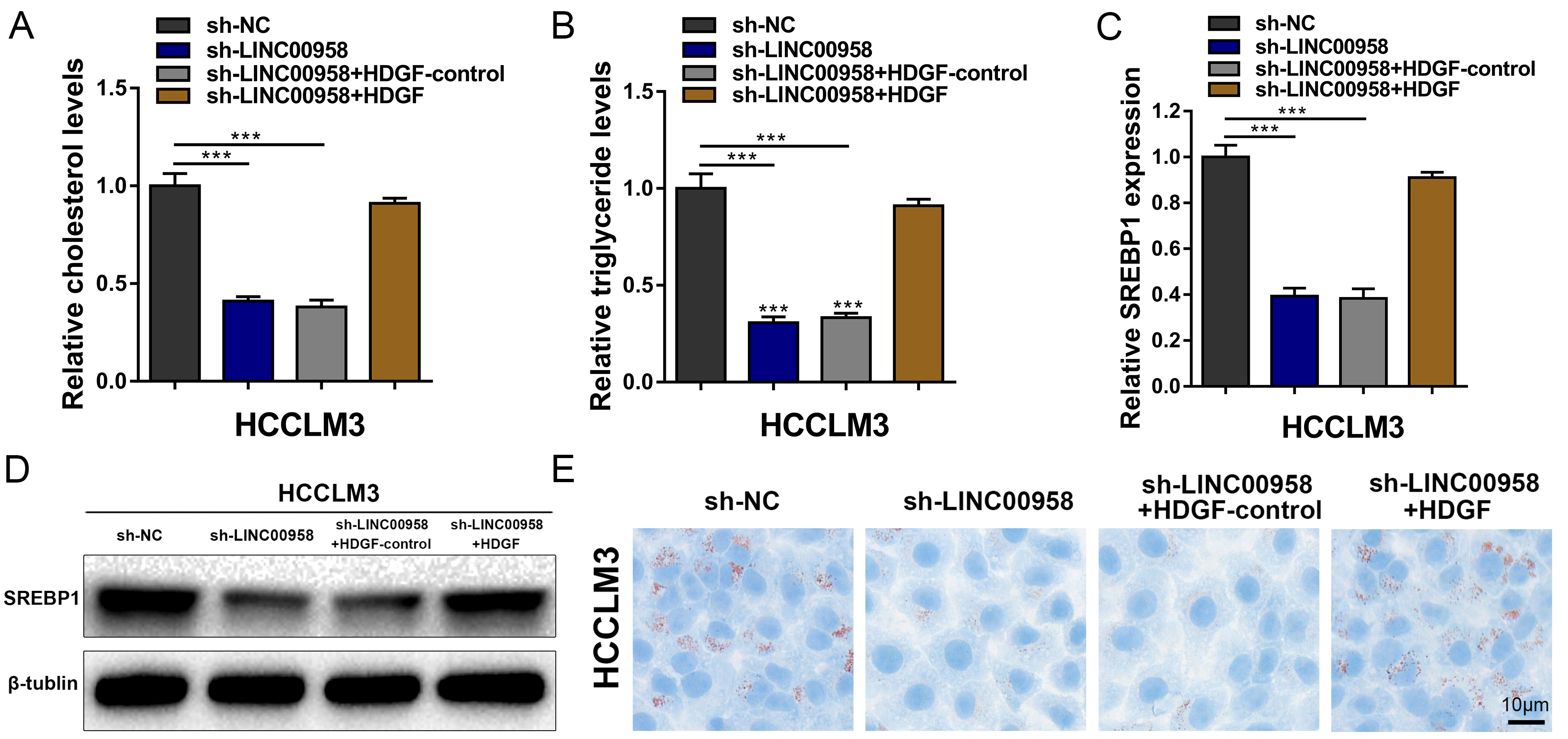

Supplement: Supplementary file 11 — Figure S6. The LINC00958/miR-3619-5p pathway modulates lipogenesis by targeting HDGF. (A) Effects of HDGF overexpression on cholesterol level in LINC00958-silenced HCCLM3 cells. The data are shown as the mean ± SEM. ***P < 0.001 vs. the sh-NC group. (B) Effects of HDGF overexpression on triglyceride level in LINC00958-silenced HCCLM3 cells. The data are shown as the mean ± SEM. ***P < 0.001 vs. the sh-NC group. (C) RT-qPCR assays were used to examine the effects of HDGF overexpression on SREBP1 level in LINC00958-silenced HCCLM3 cells. The data are shown as the mean ± SEM. ***P < 0.001 vs. the sh-NC group. (D) Western blotting was performed to investigate the effects of HDGF overexpression on SREBP1 level in LINC00958-silenced HCCLM3 cells. (E) Oil Red O staining showing the effects of HDGF overexpression on lipid droplet formation in LINC00958-silenced HCCLM3 cells. [file 13045_2019_839_MOESM11_ESM.tif]

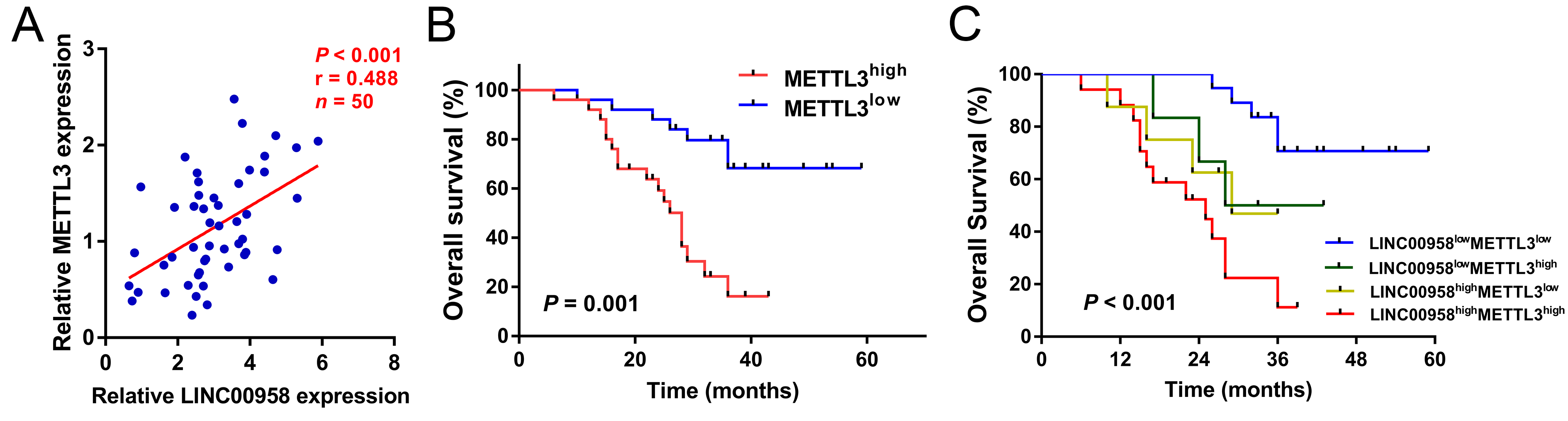

Supplement: Supplementary file 12 — Figure S7. The correlation between METTL3 expression and LINC00958 levels in HCC patient samples and its clinical impact. (A) Correlation analysis showing a positive correlation between LINC00958 and METTL3 expression (P < 0.001). (B) Kaplan-Meier survival curves showing the effect of METTL3 on overall survival (P = 0.001). (C) Kaplan-Meier survival curves showing the effect of the combination of LINC00958 and METTL3 on overall survival (P < 0.001). [file 13045_2019_839_MOESM12_ESM.tif]

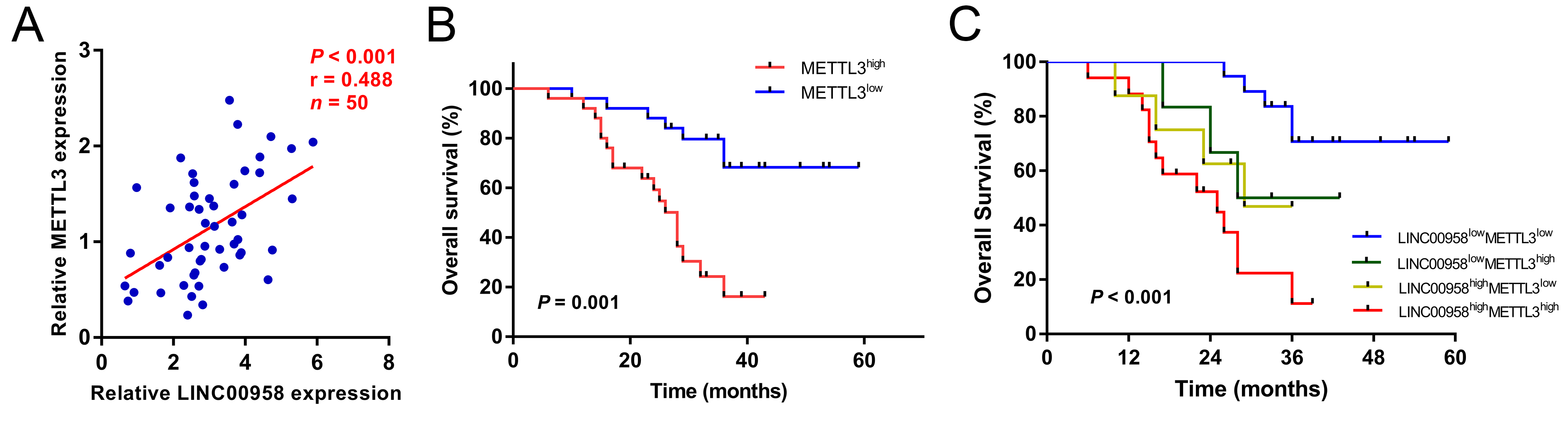

Supplement: Supplementary file 13 — Table S3. The correlation between clinicopathological characteristics and METTL3 expression level in 50 hepatocellular carcinoma patients. [file 13045_2019_839_MOESM13_ESM.tif]

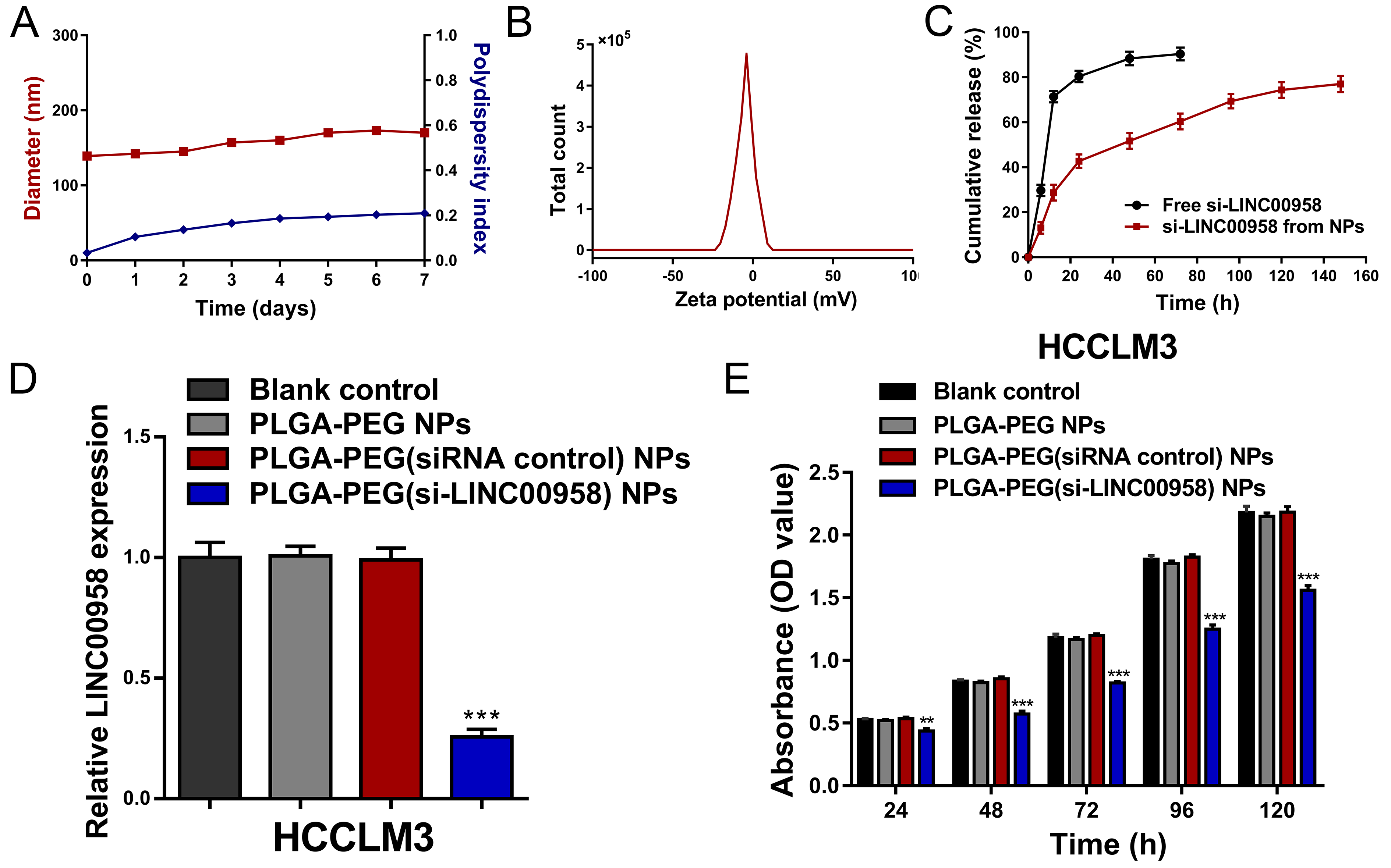

Supplement: Supplementary file 14 — Figure S8. Characterization, release behavior, and anti-proliferative capability of NPs in vitro. (A) DLS measurements of the size and PDI of PLGA-PEG(si-LINC00958) NPs for 1 week. (B) Zeta potential of PLGA-PEG(si-LINC00958) NPs. (C) In vitro release of LINC00958 siRNA from PLGA-PEG(si-LINC00958) NPs at 37 °C. (D) The knockdown efficiency of PLGA-PEG(si-LINC00958) NPs in HCCLM3 cells was evaluated using RT-qPCR. (E) CCK-8 assays were used to examine the in vitro anti-tumor capability of PLGA-PEG(si-LINC00958) NPs. **P < 0.01, ***P < 0.001. [file 13045_2019_839_MOESM14_ESM.tif]

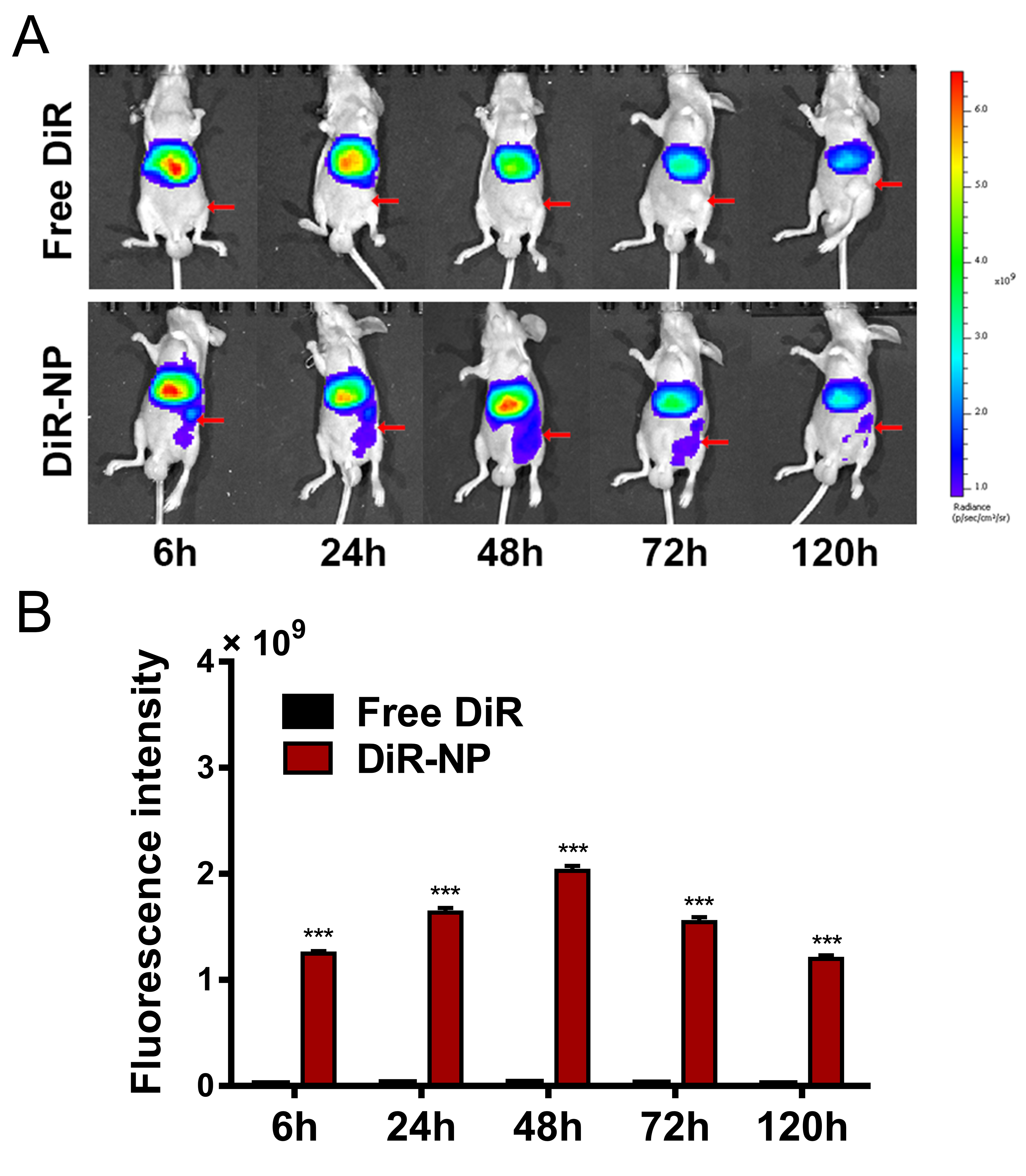

Supplement: Supplementary file 15 — Figure S9. Biodistribution of NPs by systemic injection. (A) In vivo dynamic fluorescence imaging after injecting free DiR or DiR-NP via tail vein. The xenograft tumor sites are indicated by red arrows. (B) Fluorescence intensities of the xenograft tumor sites were quantified using IVIS Lumina XRMS In Vivo Imaging System. ***P < 0.001. [file 13045_2019_839_MOESM15_ESM.tif]
