# Supplementary material for: M6A-mediated upregulation of LINC00958 increases lipogenesis and acts as a nanotherapeutic target in hepatocellular carcinoma
Source: J Hematol Oncol. 2020 Jan 8;13:5. doi: 10.1186/s13045-019-0839-x (PMC6951025; doi:10.1186/s13045-019-0839-x)
Supplement: Supplementary file 4 — Table S2. Univariate and multivariable analysis of overall survival after surgery. [file 13045_2019_839_MOESM4_ESM.docx]

**Additional file 3: Table S1.** The correlation between clinicopathological characteristics and LINC00958 expression level in 80 hepatocellular carcinoma patients.

| **Characteristics** | **No. of patients (%)** | **LINC00958 expression** | | ***P*** |
| --- | --- | --- | --- | --- |
|  |  | **Low *n* = 40 (%)** | **High *n* = 40 (%)** |  |
| Age |  |  |  | 0.108 |
| <60 years | 31 (38.8) | 19 (47.5) | 12 (30.0) |  |
| ≥60 years | 49 (61.2) | 21 (52.5) | 28 (70.0) |  |
| Gender |  |  |  | 0.431 |
| Female | 19 (23.8) | 11 (27.5) | 8 (20.0) |  |
| Male | 61 (76.2) | 29 (72.5) | 32 (80.0) |  |
| Cirrhosis |  |  |  | 0.576 |
| No | 16 (20.0) | 9 (22.5) | 7 (17.5) |  |
| Yes | 64 (80.0) | 31 (77.5) | 33 (82.5) |  |
| Hepatitis B infection |  |  |  | 0.459 |
| No | 23 (28.8) | 13 (32.5) | 10 (25.0) |  |
| Yes | 57 (71.2) | 27 (67.5) | 30 (75.0) |  |
| Differentiation |  |  |  | 0.019* |
| Well | 28 (35.0) | 19 (47.5) | 9 (22.5) |  |
| Moderate/Poor | 52 (65.0) | 21 (52.5) | 31 (77.5) |  |
| Tumor size |  |  |  | 0.025* |
| < 5 cm | 42 (52.5) | 26 (65.0) | 16 (40.0) |  |
| ≥ 5 cm | 38 (47.5) | 14 (35.0) | 24 (60.0) |  |
| Microvascular invasion |  |  |  | 0.014* |
| No | 43 (53.8) | 27 (67.5) | 16 (40.0) |  |
| Yes | 37 (46.2) | 13 (32.5) | 24 (60.0) |  |
| TNM stage |  |  |  | 0.013* |
| I/II | 35 (43.8) | 23 (57.5) | 12 (30.0) |  |
| III/IV | 45 (56.2) | 17 (42.5) | 28 (70.0) |  |
