# Supplementary material for: M6A-mediated upregulation of LINC00958 increases lipogenesis and acts as a nanotherapeutic target in hepatocellular carcinoma
Source: J Hematol Oncol. 2020 Jan 8;13:5. doi: 10.1186/s13045-019-0839-x (PMC6951025; doi:10.1186/s13045-019-0839-x)
Supplement: Supplementary file 6 — Supplementary Material 1. miRNAs with complementary sequences to LINC00958 predicted by starBase and miRDB. [file 13045_2019_839_MOESM6_ESM.docx]

**Additional file 13: Table S3.** The correlation between clinicopathological characteristics and METTL3 expression level in 50 hepatocellular carcinoma patients.

| **Characteristics** | **No. of patients (%)** | **METTL3 expression** | | ***P*** |
| --- | --- | --- | --- | --- |
|  |  | **Low *n* = 25 (%)** | **High *n* = 25 (%)** |  |
| Age |  |  |  | 0.571 |
| <60 years | 24 (48.0) | 11 (44.0) | 13 (52.0) |  |
| ≥60 years | 26 (52.0) | 14 (56.0) | 12 (48.0) |  |
| Gender |  |  |  | 0.765 |
| Female | 17 (34.0) | 9 (36.0) | 8 (32.0) |  |
| Male | 33 (66.0) | 16 (64.0) | 17 (68.0) |  |
| Cirrhosis |  |  |  | 0.508 |
| No | 12 (24.0) | 7 (28.0) | 5 (20.0) |  |
| Yes | 38 (76.0) | 18 (72.0) | 20 (80.0) |  |
| Hepatitis B infection |  |  |  | 0.370 |
| No | 17 (34.0) | 10 (40.0) | 7 (28.0) |  |
| Yes | 33 (66.0) | 15 (60.0) | 18 (72.0) |  |
| Differentiation |  |  |  | 0.002* |
| Well | 27 (54.0) | 19 (76.0) | 8 (32.0) |  |
| Moderate/Poor | 23 (46.0) | 6 (24.0) | 17 (68.0) |  |
| Tumor size |  |  |  | 0.018* |
| < 5 cm | 18 (36.0) | 13 (52.0) | 5 (20.0) |  |
| ≥ 5 cm | 32 (64.0) | 12 (48.0) | 20 (80.0) |  |
| Microvascular invasion |  |  |  | 0.023* |
| No | 22 (44.0) | 15 (60.0) | 7 (28.0) |  |
| Yes | 28 (56.0) | 10 (40.0) | 18 (72.0) |  |
| TNM stage |  |  |  | 0.001* |
| I/II | 28 (56.0) | 20 (80.0) | 8 (32.0) |  |
| III/IV | 22 (44.0) | 5 (20.0) | 17 (68.0) |  |
